# Mitochondrial genomic variation associated with higher mitochondrial copy number: the Cache County Study on Memory Health and Aging

**DOI:** 10.1186/1471-2105-15-S7-S6

**Published:** 2014-05-28

**Authors:** Perry G Ridge, Taylor J Maxwell, Spencer J Foutz, Matthew H Bailey, Christopher D Corcoran, JoAnn T Tschanz, Maria C Norton, Ronald G Munger, Elizabeth O'Brien, Richard A Kerber, Richard M Cawthon, John SK Kauwe

**Affiliations:** 1 Department of Biology, Brigham Young University, Provo, UT, USA; 2 ARUP Institute for Clinical and Experimental Pathology, Salt Lake City, UT, USA; 3 Human Genetics Center, University of Texas School of Public Health, Houston, TX, USA; 4 Department of Mathematics and Statistics, Utah State University, Logan, UT, USA; 5 Center for Epidemiologic Studies, Utah State University, Logan, UT, USA; 6 Department of Psychology, Utah State University, Logan, UT, USA; 7 Department of Family Consumer and Human Development, Utah State University, Logan, UT, USA; 8 Department of Nutrition, Dietetics, and Food Sciences, Utah State University, Logan, UT, USA; 9 Department of Epidemiology and Population Health, University of Louisville, Louisville, KY, USA; 10 Department of Human Genetics, University of Utah, Salt Lake City, UT, USA

## Abstract

**Background:**

The mitochondria are essential organelles and are the location of cellular respiration, which is responsible for the majority of ATP production. Each cell contains multiple mitochondria, and each mitochondrion contains multiple copies of its own circular genome. The ratio of mitochondrial genomes to nuclear genomes is referred to as mitochondrial copy number. Decreases in mitochondrial copy number are known to occur in many tissues as people age, and in certain diseases. The regulation of mitochondrial copy number by nuclear genes has been studied extensively. While mitochondrial variation has been associated with longevity and some of the diseases known to have reduced mitochondrial copy number, the role that the mitochondrial genome itself has in regulating mitochondrial copy number remains poorly understood.

**Results:**

We analyzed the complete mitochondrial genomes from 1007 individuals randomly selected from the Cache County Study on Memory Health and Aging utilizing the inferred evolutionary history of the mitochondrial haplotypes present in our dataset to identify sequence variation and mitochondrial haplotypes associated with changes in mitochondrial copy number. Three variants belonging to mitochondrial haplogroups U5A1 and T2 were significantly associated with higher mitochondrial copy number in our dataset.

**Conclusions:**

We identified three variants associated with higher mitochondrial copy number and suggest several hypotheses for how these variants influence mitochondrial copy number by interacting with known regulators of mitochondrial copy number. Our results are the first to report sequence variation in the mitochondrial genome that causes changes in mitochondrial copy number. The identification of these variants that increase mtDNA copy number has important implications in understanding the pathological processes that underlie these phenotypes.

## Background

Mitochondria are the location of the citric acid or Krebs Cycle, which produces the majority of ATP for cellular work. Each cell has multiple mitochondria and each mitochondrion contains one or more copies of its own circular genome (mtDNA), which is 16569 bases in length and encodes 37 genes. Mitochondria are necessary for survival and malfunctioning mitochondria are the cause of a variety of diseases [1-11]. Mitochondrial diseases tend to affect the CNS or muscle tissue because of the high energy needs of these tissues [12]. Mitochondrial diseases have been well studied and can be the result of genetic variation in the mitochondrial and/or nuclear genomes. Pathogenic nuclear mutations are inherited in a typical Mendelian pattern and can present with a dominant, recessive, or X-linked dominant or recessive inheritance pattern. Examples of mitochondrial diseases caused by mutations in the nuclear genome include Friedrich's ataxia [13], Wilson's disease [14], and Barth syndrome [15].

In contrast, mitochondrial diseases caused by variation in the mtDNA are not as straightforward. Mitochondria are maternally inherited, so mitochondrial disease caused by these variants will display maternal inheritance. However, in most cases both normal and pathogenic mtDNA are inherited together and the mix can vary from predominantly wild type to predominantly pathogenic. Depending on the severity of the mutation, proportion of wild type versus affected mitochondria, and the specific tissue, there may or may not be a disease phenotype. Over the course of life the proportion of diseased mitochondria can change, possibly reaching a critical threshold at which the disease phenotype is expressed. Alternatively, a constant proportion of diseased mitochondria might contribute to disease only when present in combination with one or more additional factors (e.g. stresses of various kinds, and/or aging). In addition to inherited mtDNA variation, mtDNA is prone to somatic mutations [16], and if affected mtDNA are propagated they can eventually reach a threshold at which mitochondrial function is insufficient to support normal cellular functions and disease appears. Some examples of disorders caused by mtDNA mutations are Kearns-Sayre syndrome [8], diabetes mellitus and deafness [7], Leber's hereditary optic neuropathy [9], Leigh Syndrome [11], and Myoclonic Epilepsy with Ragged Red Fibers (a.k.a. MERRF syndrome) [10].

Additionally, mitochondria have a role in aging. The free-radical theory of aging, or mitochondrial free radical theory of aging, hypothesizes that aging occurs as damage from reactive oxygen species (ROS) accumulates. ROS are produced in the electron transport chain [17] and readily oxidize DNA and RNA, amino acids, and fatty acids [18-20]. Damage from ROS can accumulate with time resulting in cellular dysfunction, and death [21].

MtDNA copy number, or the cellular ratio of mitochondrial genomes to nuclear genomes, decreases with age in some, but not all, tissues [22-25] and mtDNA copy number variation has been associated with numerous phenotypes [26-38]. MtDNA copy number is tissue dependent [39] and varies with age and the energy needs of the cell [24,25,40].

Several nuclear genes regulate mtDNA copy number. First, there is substantial evidence that mitochondrial transcription factor A (*TFAM*) regulates mtDNA copy number [41-44]. The *Mec1*/*Rad53 *(yeast) pathway has been implicated in controlling mtDNA copy number, and mtDNA levels can be controlled by any of several genes responsible for regulating the pathway [45]. *p53 *deficient cells or mutated *p53 *leads to decreased levels of mtDNA [46]. Two common nuclear SNPs in signal transducer and activator of transcription 3 (*STAT3*) were significantly associated with mtDNA levels in leukocytes [47]. Both the Ras pathway and *p66Shc *likely have roles in regulating mtDNA copy number [48]. *MnSOD *prevents decreases in mtDNA levels by preventing a decrease in mtDNA replication proteins [49]. And finally, overexpression of *Twinkle *increases mtDNA copy number [50].

The direct role for the mitochondrial genome regulating levels of mtDNA has not been studied extensively. Here we conduct a genetic association study of full mitochondrial genome data and mtDNA copy number in individuals from the Cache County Study on Memory Health and Aging. Our results identify association between mitochondrial haplogroups U5A1 and T2 and increased mtDNA copy numbers.

## Methods

### Ethics statement

As described in [51], all study procedures were approved by the Institutional Review Boards of Brigham Young University, Utah State University, Duke University, and Johns Hopkins University. Written consent was obtained for each individual. To verify a subject's capacity to consent, subjects attempted the Modified Mini-Mental State Exam (3MS). If there was an indication of poor cognitive ability as determined by poor performance on the entire test (scoring below a designated total of 60 points), poor performance on temporal or spatial orientation, or clear difficulty in understanding the nature of the interview, the visit was discontinued and informed consent was obtained from a responsible caregiver- often the next-of-kin. We re-consented subjects/caregivers at each study visit and procedure.

### Sample acquisition and sequencing

Samples for this study were selected from the Cache County Study on Memory Health and Aging [52]. This study was initiated in 1994 to investigate associations of genetic and environmental factors with cognitive function. In 1994, the 5,092 individuals enrolled in the study from Cache County, Utah, represented 90% of all Cache County, Utah, residents who were 65 or older. The cohort was followed for 12 years and data (medical histories, demographics, and a multistage dementia assessment) were collected in four triennial waves. The Utah population is similar to other U.S. populations of northern European ancestry characterized by very little inbreeding. The founding group of Utah's population was unrelated and migrated from various locations in Europe [53-55].

The Utah Population database (UPDB) has complete pedigree information going back 14 generations to the original Utah Founders. Using this information we identified individuals from the Cache County Study with the same maternal line of inheritance (matrilineage). We randomly selected one individual from each matrilineage, selecting individuals from the largest matrilineages first to maximize our ability to infer mitochondrial genomic information. Given our resources, we were able to sequence a representative sample from 274 of the 3151 matrilineages that exist in the Cache County Study samples. The sequenced mitochondrial genomes represent many different major mitochondrial haplogroups (Table 1). 287 samples were sent to Family Tree DNA (http://www.familytreedna.com) for Sanger sequencing of the mitochondrial genomes. Two samples failed quality control at Family Tree DNA. Based on maternal inheritance of the mtDNA we inferred that individuals who share matrilineal relationships have the same mtDNA. Using this we inferred the status of full mitochondrial genome sequence for 722 additional individuals for a total of 1007 individuals, not accounting for de novo mutation. The extensive pedigree data in the UPDB allows identification of shared maternal lineages for very distant relationships. As this was a population-based study it is one generation in depth, but there are extended familial relationships, even very distant cousins. Ridge et al [51] contains additional details about the sequencing and inference of the mtDNA status in this dataset.

**Table 1:** Distribution of major mtDNA haplogroups/clusters.

Major Haplogroup	Number	**Ethnicities**[94,95]
H	424	European
U	147	European
T	121	European
J	99	European
K	95	European
V	34	European
I	21	European
W	20	European
HV	18	European
X	8	European
C	5	Asian
L	4	African
Missing^1^	11	

Here we report the number of individuals belonging to each of the major haplogroups represented in our dataset along with case-control status. This Table first appeared in [51]. ^1^Missing major haplogroup

### Measurement of mtDNA copy number

Relative quantitation of the ratio of the copy number of the mitochondrial genome to the copy number of the nuclear single copy gene beta-globin, as compared to that ratio in a reference DNA sample, was determined by monochrome multiplex quantitative polymerase chain reaction (QPCR). Buccal sample cell lysates were diluted in water (containing yeast total RNA as carrier, at 2.5 ng per microliter) to a final total cellular DNA concentration of approximately 1 ng per 10 microliters. QPCR was carried out in 25 microliter reactions, containing 10 microliters of the diluted buccal lysate and 15 microliters of QPCR reagent mix with primers.

The QPCR reagent mix, without primers, was exactly as described by Cawthon [56]. The primers for mtDNA amplification were mt3257u, 5'- GCAGAGCCCGGTAATCGCA-3', and mt3272d, 5'-TAAGAAGAGGAATTGAACCTCTGACTGTAA-3'. The mt3272d primer has previously been shown to be specific to mtDNA and unable to amplify any nuclear-embedded mtDNA-like sequences (numts) from rho 0 cell line DNA [57] (Rho 0 cell lines are mtDNA-free). The primers for the beta-globin gene were hbgugc2, 5'-CGGCGGCGGGCGGCGCGGGCTGGGCGGCTTCATCCACGTTCACCTTG-3', and hbgdgc2, 5'-GCCCGGCCCGCCGCGCCCGTCCCGCCGGAGGAGAAGTCTGCCGTT-3'. Both beta-globin primers contained 5' GC-clamp (non-templated) sequences that confer a high melting temperature on their amplicon. Each of the four primers was present at a final concentration of 900 nM.

The thermal profile for QPCR began with 95 degrees C for 15 minutes to activate the hot-start polymerase and fully denature the DNA; followed by 35 cycles of: 94 degrees for 15 sec, 62 degrees for 20 sec, 72 degrees for 15 sec with signal acquisition (to read the mtDNA amplification signal), 84 degrees for 10 sec, and 88 degrees for 15 sec (to read the beta-globin signal). In this monochrome multiplex QPCR (MMQPCR) strategy, first described by Cawthon [56], the higher copy number target (in this case mtDNA) has its amplification signal collected over a cycle range in which the lower copy number target's (in this case the beta-globin genes) amplification signal is still at baseline, and the lower copy number target's amplificaton signal is collected in later cycles, at a temperature that is sufficiently high to completely melt the amplicon of the higher copy number target, driving its signal to baseline so that the signal from the high melting amplicon can be cleanly read. All QPCR runs were done on Bio-Rad MyiQ real-time machines, using the manufacturer's accompanying software. The Standard Curve method for relative quantitation was used, with 36 ng of a reference DNA sample as the high end, and four additional standard concentrations obtained via 3-fold serial dilutions from the high end. Each subject's buccal lysate was assayed in triplicate. The average of the three measurements for each sample was used in this study (Additional File 1). DNA is not available from other tissue for the majority of these samples.

### Sequence and statistical analyses

We used ClustalW [58] to align the mitochondrial genomes and inferred a haplotype network using TCS [59] and the 285 sequenced mitochondrial genomes. In a haplotype network, segments of branches correspond to a single sequence feature (single nucleotide variant, indel, etc.), and nodes in the network correspond to haplotypes. Branches, comprised of one or more segments, connect observed nodes, while clades are comprised of one or more observed nodes, and are defined by a branch.

Genotype-phenotype associations were evaluated using an evolution-based method known as TreeScanning [60,61] that makes use of haplotype networks. Haplotype networks provide a framework from which to select evolutionarily related haplotypes to pool together for comparison. Additional details about the application of TreeScanning to this dataset can be found in Ridge et al [51]. The null hypothesis of TreeScanning is that the phenotype does not differ in distribution across the genotypes derived from allelic classes defined by the branches of the haplotype network. Each branch partitions the haplotypes into bi-allelic pools from which genotypes are constructed and treated as a separate test. Because we have multiple tests that are correlated we obtained multiple-test corrected p-values by a permutation analog of the sequential step-down Bonferroni [62] with 10,000 permutations. If significant branches are found in the first round of TreeScanning, a second round of TreeScanning is performed that can detect phenotypic heterogeneity within the allelic classes of the significant branch. This is accomplished by creating a three-allele system and using conditional permutations that hold one of the alleles constant while subdividing the other class into two alleles [60]. Significant branches define clades.

For these analyses we tested for association with mitochondrial copy number after adjusting for gender, age, and familial relationships. Familial adjustment scores, which quantify the variance in mtDNA copy number that is due to familial relationships between individuals in the dataset, were computed using the method developed by Kerber (modified for a continuous trait) [63]. For each individual we summed the products of the mtDNA copy number and the pairwise kinship coefficient (a pairwise measure of relatedness) with each of the other individuals in the sample. This sum is then divided by the total number of samples in the dataset. Finally, we divide by the mtDNA copy number of the individual, yielding a value, which represents the relationship between mtDNA copy number and relatedness to other individuals in the dataset. We calculated familial adjustment scores for each individual in the dataset using the following equation:

$$\text{familial adjustment score = }\frac{\frac{\sum_{j=1}^{N}copy\;number\;j*f\left(individual,j\right)}{N}}{individual\;copy\;number}$$

Where *N *is the number of individuals in the cohort and *f(individual, j) *is the kinship coefficient between the individual for whom we are calculating a familial adjustment score (labeled as 'individual' in the formula) and individual *j *(representing each of the other individuals in the dataset one at a time). Inclusion of this score as a covariate in our analyses removes variance in mtDNA copy number that is due to relatedness between individuals, making it possible to test for association independently of pedigree relationships in the data. This adjustment addresses both maternal and paternal relationships in the data, thus correcting for possible nuclear genomic confounds as well. Each analysis was performed with 10,000 permutations. Only tests with at least two relevant genotypic classes, each containing five or more individuals, were tested. Significance was inferred if the multiple-test-corrected p-value was less than 0.05.

### Bioinformatic analyses of variants

In order to determine the functional impact of variants of interest we applied *in silico *functional prediction algorithms, analyzed pathways, examined protein sequence conservation, and identified conserved domains. We obtained protein sequences from NCBI using blast [64], aligned and analyzed them using the CLCViewer (http://clcbio.com/), identified conserved domains using the NCBI conserved domain database [65], identified pathways using Ingenuity (http://Ingenuity.com/), and obtained functional predictions from polyphen-2 [66] and SIFT [67,68] webservers. In each case we used default settings.

## Results

### Haplotype network and mtDNA variation

We sequenced 285 complete mitochondrial genomes from individuals in the Cache County Study on Memory Health and Aging and imputed 722 additional full mitochondrial genomes using maternal lineages for a full dataset of 1007 full mitochondrial genomes. We built our network using the 285 genotyped individuals (Additional Files 2, 3). Our network contained 249 different haplotypes and the majority of haplotypes (152 of 249) were observed in three or fewer individuals with the two most frequently observed haplotypes observed in 39 and 32 individuals, respectively. Our network contained one unresolved loop and the ambiguity was factored into subsequent analyses.

We identified 899 single nucleotide variants (SNVs), 26 insertions, and 20 deletions in our dataset. The most frequently observed SNVs occurred in 281 genomes (m.263A>G, m.8860A>G, and m.15326A>G), and three more SNVs were observed in 280 genomes (m.750A>G, m.1438A>G, and m.4769A>G). Compared to the reference sequence (NC_012920), each person had an average of 25.3 variants (52 variants were the most identified in a single individual and 2 variants the fewest, each extreme observed in one person).

The distribution of major mitochondrial haplogroups within our dataset is reported in Table 1 (major mitochondrial haplogroups/clusters) and Additional File 4 (major mitochondrial haplogroups and sub-haplogroups). Our dataset contained individuals from 102 major mitochondrial haplogroups/clusters (or sub-haplogroups) in our dataset. As expected, the majority (987 of 1007) of individuals in our dataset belonged to European-based major mitochondrial haplogroups. We identified three different branches, corresponding to two different clades, significantly associated with mtDNA copy number.

### Branches 124 and 121 are associated with mtDNA copy number

**Figure 1:**
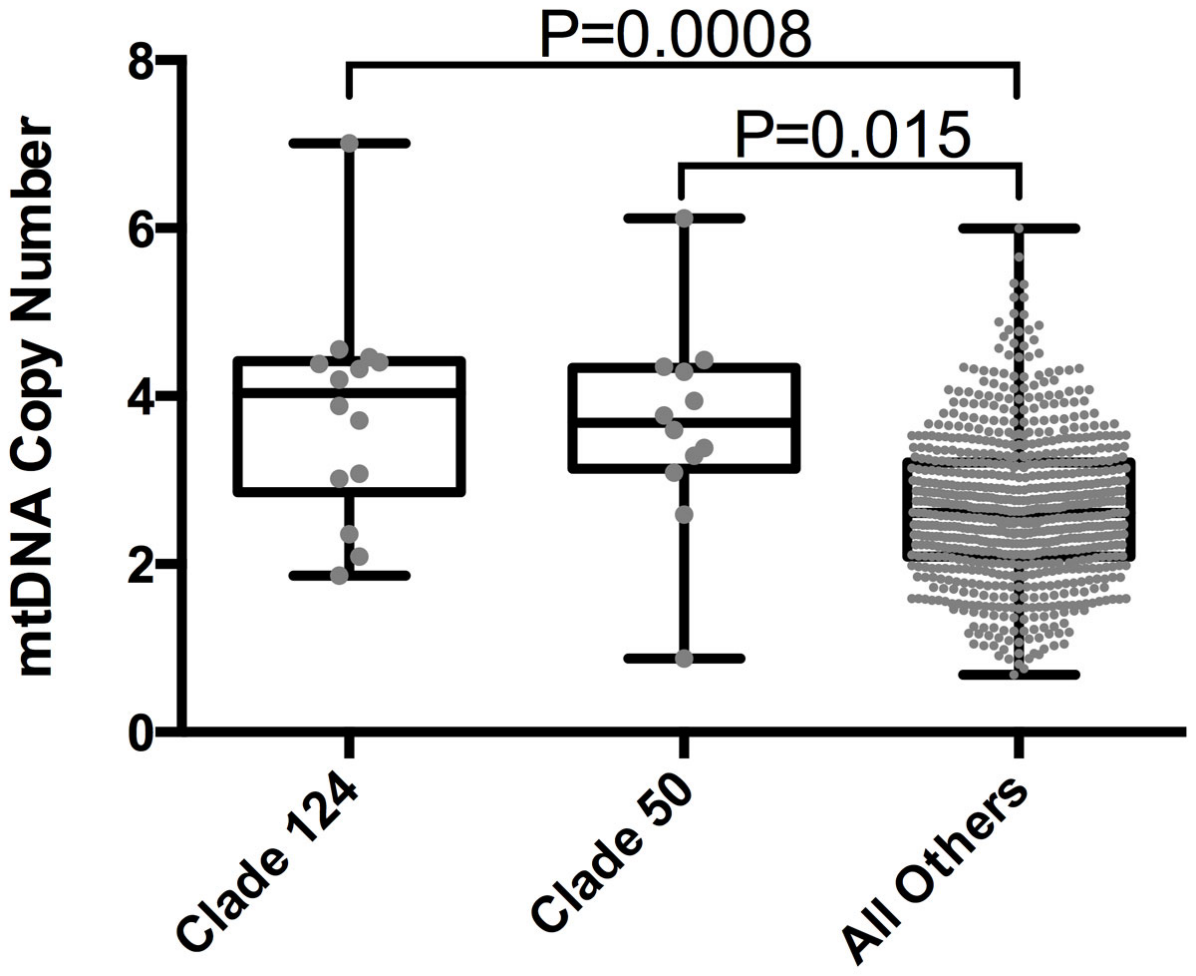
**Box plot comparing mitochondrial copy number between different clades**. The grey dots represent the mitochondrial copy number for each member of the representative groups. The top and bottom of the boxes correspond to the 75^th ^and 25^th ^percentiles, respectively, and the line through the box is the median mitochondrial copy number for the group. The whiskers correspond to the maximum and minimum mitochondrial copy numbers for the group. Three different groups are represented here: the clades defined by branches 124 and 50, and a group containing all other individuals in the dataset. The y-axis is the mitochondrial copy number. The reported p-values are corrected.

**Figure 2:**
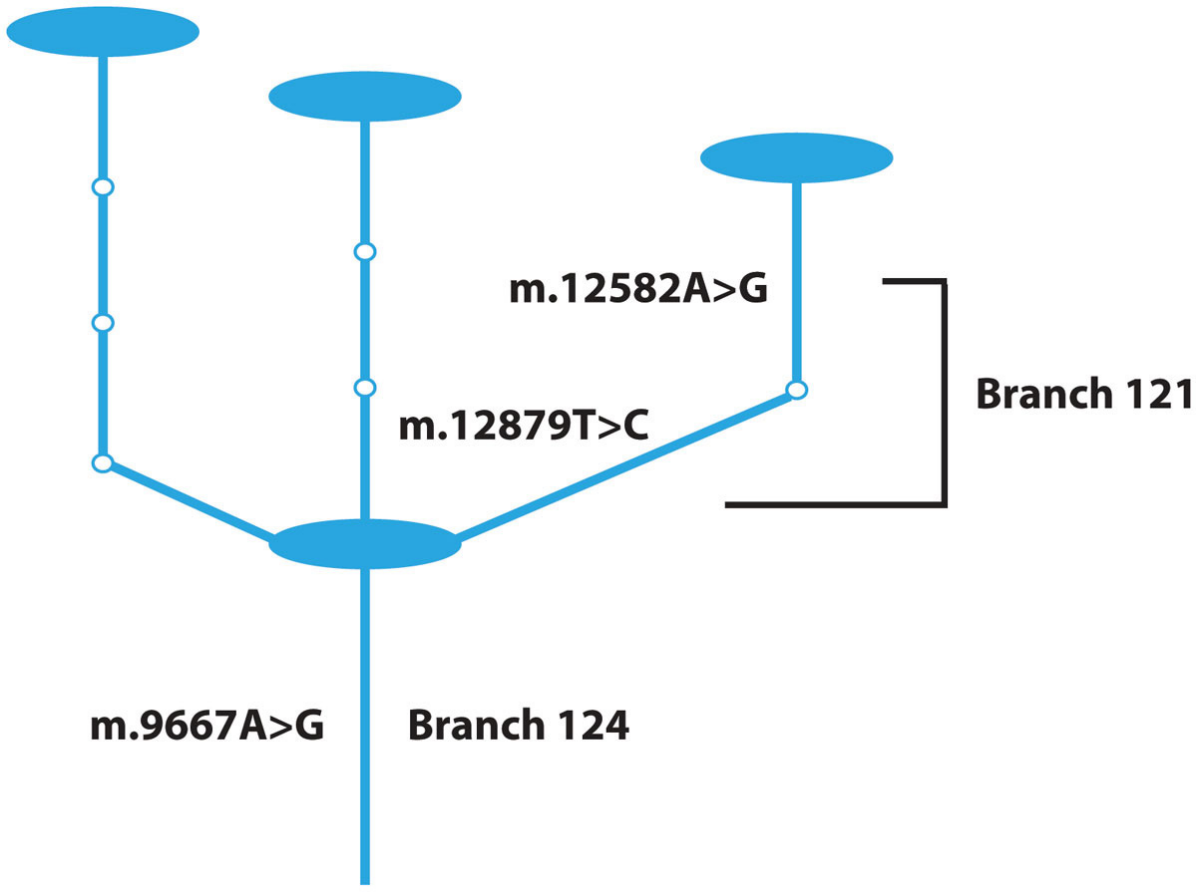
**Significant branches**. This is a subset of the full haplotype network (Additional File 2), focused on the two significant clades defined by branches 124 and 121, which are labeled here. The blue ovals represent haplotypes observed in our dataset, and the smaller white circles are unobserved haplotypes. Only the variants that define branches 124 and 121 are labeled.

First, branches 124 and 121, p-values of 8.0e-4 and 0.0043 (multi-test corrected p-values), respectively (Table 2, Figure 1), were associated with higher mtDNA copy number. The clade defined by branch 121 is wholly contained within branch 124 (Figure 2); therefore, these two branches are highly correlated and represent the same effect. Branch 124 is defined by a single variant (Table 3), *m.9667A>G*. This is a missense variant, *p.Asn154Ser*, located in cytochrome C oxidase 3 (*COXIII*). Branch 121 is defined by two variants (Table 3), *m.12582A>G *and *m.12879T>C*, both synonymous variants in NADH dehydrogenase 5 (*ND5*).

**Table 2:** Demographic information for significant contrasts.

	Individuals/Missing	p-value^1^		p-value^2^		Age	Male/Female	Mean copy #
		**Nominal**	**Corrected**	**Nominal**	**Corrected**			

Whole network	1007/193	N/A	N/A	N/A	N/A	75.6	442/565	2.69

Branch 124	17/3	0	6.0e-4	0	8.0e-4	75.2	9/8	3.81

Branch 121	10/1	0	0.002	1.0e-4	0.0043	76.3	4/6	4.01

Branch 50	15/3	2.0e-4	0.017	2.0e-4	0.015	78.4	7/8	3.64

Here we report demographic information for each of the significant contrasts and for all the individuals in the dataset. The clade represented by Branch 121 is wholly contained within Branch 124, so these two contrasts represent a single effect. Branches 124 and 50 represent separate effects. Missing refers to the number of individuals for whom we have no mtDNA copy number measurement.^1^p-values were calculated controlling only for the other significant branches ^2^p-values were calculated using age, gender, mtDNA copy number family risk score, and the other significant effects as covariates

**Table 3:** Defining variants for the three significant contrasts.

Branch	Nucleotide Change	Amino Acid Change	Gene
Branch 124	m.9667A>G	p.Asn154Ser	Cytochrome C Oxidase 3

Branch 121	m.12582A>G	p.Leu82Leu	NADH Dehydrogenase 5

	m.12879T>C	p.Gly181Gly	NADH Dehydrogenase 5

Branch 50	m.5277T>C	p.Phe270Leu	NADH Dehydrogenase 2

	m.5426T>C	p.His319His	NADH Dehydrogenase 2

	m.6489C>A	p.Leu196Ile	Cytochrome C Oxidase 1

	m.8270C>T	N/A	Intergenic

	m.del8281-8289	N/A	Intergenic

	m.14458C>T	p.Ala72Ala	NADH Dehydrogenase 6

	m.15028C>A	p.Leu94Leu	Cytochrome B

	m.15043G>A	p.Gly99Gly	Cytochrome B

There were three significant contrasts in our dataset, two of which, 124 and 121, which represent a single effect. One or more sequence features define each of the branches, and each is listed here with the resulting protein change, and the gene the feature is located in.

Since these two branches correspond to a single effect and branch 121 is wholly contained within branch 124, we consider only the clade defined by branch 124 from this point forward. This clade contains 14 individuals for whom we have mtDNA copy number measurements. Pairwise kinship coefficients are reported for these individuals in Additional File 5. Individuals in this clade have a mtDNA copy number nearly 50% higher (3.81 compared to 2.69, p-value 8.0e-4) than individuals in the rest of the dataset.

All of the individuals in the clade defined by branch 124 belong to major mitochondrial haplogroup U5A1, and have one of four different haplotypes (represented by nodes in Figure 2). Nine other individuals (five different haplotypes) in the dataset also belong to U5A1. These individuals are located in adjacent clades to the one defined by branch 124 and have significantly lower mitochondrial copy numbers than the other U5A1 individuals (p-value 0.0082). The contrast of all U5A1 individuals against the rest of the dataset was nominally significant (p-value 0.0019). While no d-loop variants define branch 124, *m.16399A>G*, a d-loop variant, is only found in the U5A1 individuals in our dataset and in general appears to be found in all U5A1 individuals [69].

### Branch 50 is associated with mtDNA copy number

Branch 50 is the third branch significantly associated higher mtDNA copy number (p-value 0.015, multi-test corrected p-value, Table 2 Figure 1). This represents a statistically separate effect as we controlled for the effect of branch 124 in our analyses (just as we controlled for branch 50 in our analyses of branch 124). Eight sequence features define branch 50: seven single nucleotide variants and one nine base pair deletion (Table 3). Six of the eight features are intergenic or synonymous, but the other two are both missense variants. *m.5277T>C *(*p.Phe270Leu*) is a missense variant in NADH dehydrogenase 2 (*ND2*) and *m.6489C>A *(*p.Leu196Ile*) is a missense variant in cytochrome C oxidase 1 (*COXI*).

In the clade defined by branch 50 there are 12 individuals with mtDNA copy number measurements. Pairwise kinship coefficients are reported for these individuals in Additional File 6. The average mtDNA copy number for individuals in this clade is 3.64 and is significantly higher than the average for the rest of the dataset (2.69, p-value 0.015). Individuals in this clade belong to major mitochondrial haplogroup T2 and all have the exact same haplotype. There were no other T2 individuals in the rest of our dataset; however, there were T2A, T2B, T2C, and T2E individuals. The contrast between T2 and all T2 sub-haplogroups (T2A, T2B, T2C, and T2E) and the rest of the data was nominally significant, p-value 0.019, and the contrast of T2B individuals alone against the rest of the dataset was nominally significant, p-value 0.0062.

### Bioinformatic Analyses of m.9667A>G, branch 124

**Figure 3:**
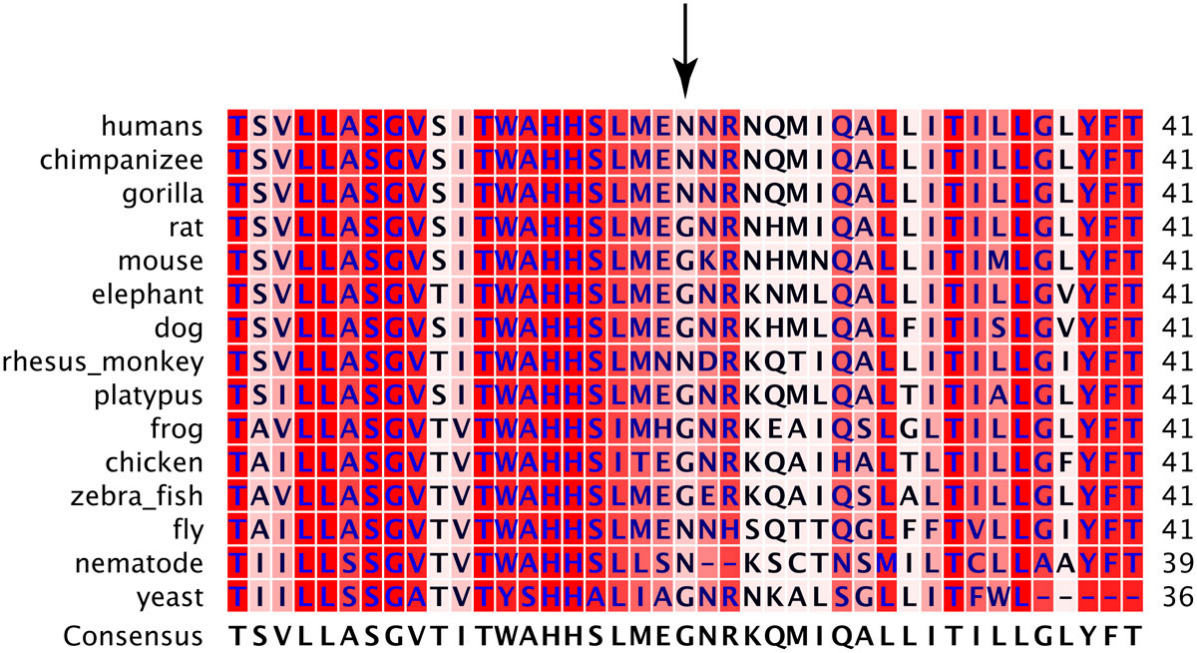
**Multiple sequence alignment of COXIII**. Position 21 in the alignment corresponds to position 154 in COXIII. Background colors correspond to the level of conservation of that position in the alignment. The darker the shade of red, the higher the conservation.

*m.9667A>G *is the defining sequence change between the U5A1 individuals in our dataset who had significantly higher mtDNA copy number levels from the other U5A1 individuals in our dataset whose copy number measurements were not statistically different from the rest of the dataset. *m.9667A>G *causes an amino acid substitution, asparagine to serine, at position 154 of *COXIII*, which is located in an 11 residue stretch between transmembrane domains. Since this is a missense mutation, we sought to determine if it changes or inhibits *COXIII *and/or the cytochrome c oxidase complex. We compared *COXIII *sequences in organisms from humans through yeast by aligning a 41-residue stretch of *COXIII*. In Figure 3, position 154 of *COXIII *(the position of the amino acid substitution corresponding to *m.9667A>G*) is in position 21 of the alignment. As seen in Figure 3, two different amino acids appear in this position: asparagine and glycine. Asparagine and glycine are both uncharged amino acids; however, asparagine is polar, whereas glycine is nonpolar. M.9667A>G results in serine replacing asparagine. Serine is polar and similar in size to asparagine (asparagine 132.1 g/mol, glycine 75.1 g/mol, and serine 105.1 g/mol).

We further analyzed the effect of this substitution on *COXIII *by using in silico algorithms that predict the effect of amino acid substitutions on protein function using a variety of criteria such as conservation, amino acid biochemical properties, known domains/structures of the protein, etc. Polyphen-2 predicted the substitution to be benign and SIFT predicted a pathogenic mutation, but noted that its prediction was of very low confidence.

**Figure 4:**
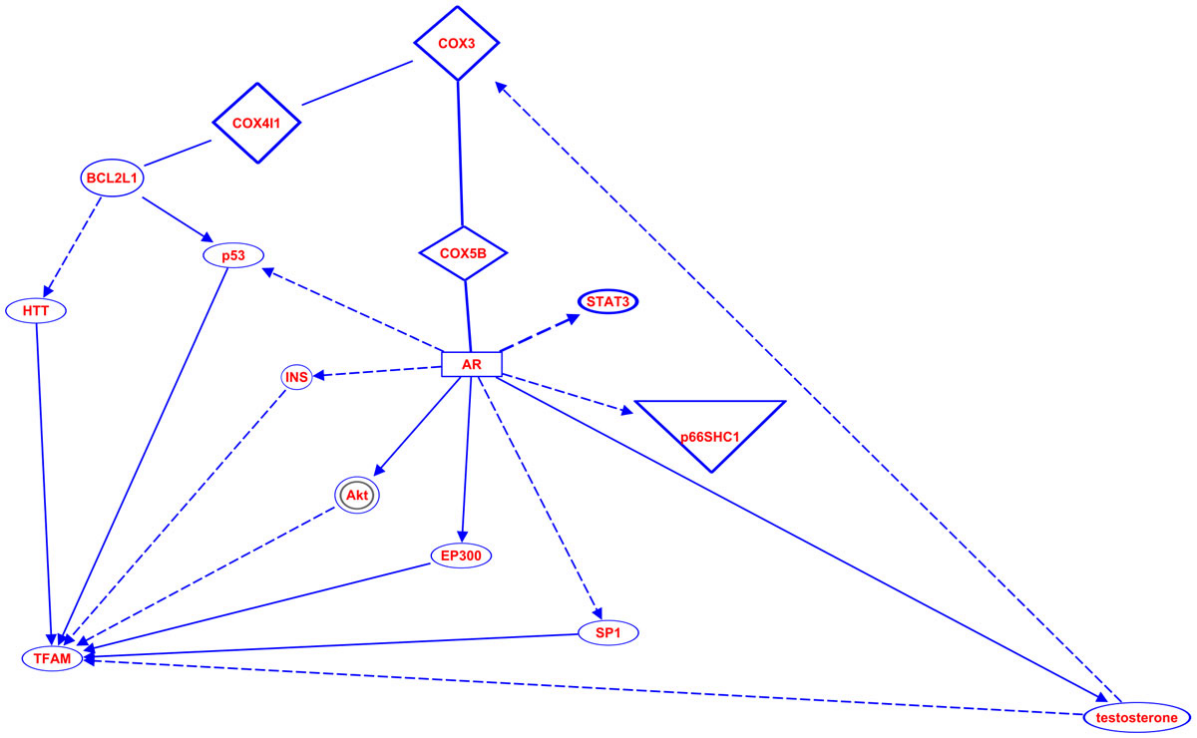
**Pathways between COXIII and known regulators of mtDNA copy number**. Here we show all the known pathways between COXIII and the different genes known to regulate or modify mtDNA copy number.

Lastly, we looked at possible interactions of *COXIII *with known regulators (listed in the Introduction) of mtDNA copy number to identify mechanisms *m.9667A>G *could cause the increased copy number. We found common regulators of both *COXIII *and the mtDNA copy number regulators, and we found ways that these regulators could affect *COXIII *expression; however, we identified no pathways by which *COXIII *could regulate mtDNA copy number by known mechanisms (Figure 4).

### Bioinformatic Analyses of m.5277T>C and m.6489C>A, branch 50

It is more difficult to say which variants are causing the increase in mtDNA copy number for the clade-defined by branch 50 since this branch consists of eight different sequence features. We chose to focus our analyses on two of the features: *m.5277T>C *and *m.6489C>A *since these two variants are missense variants and the six others features are either synonymous or intergenic changes.

**Figure 5:**
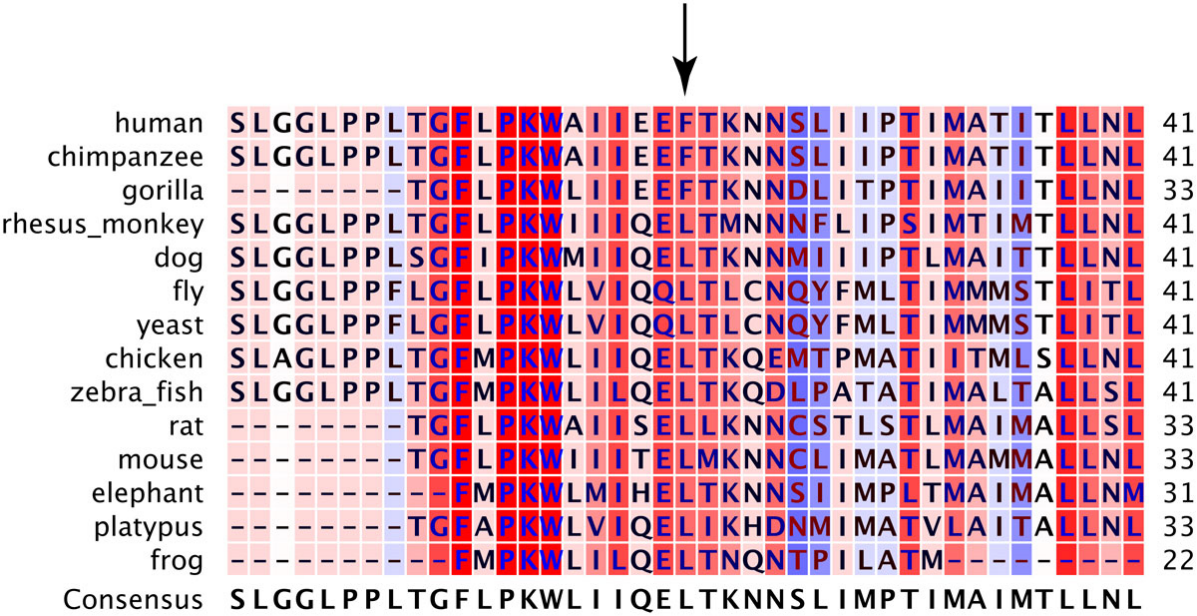
**Multiple sequence alignment of ND2**. Position 21 in the alignment corresponds to position 270 in ND2. Background colors are as described in Figure 3.

First, *m.5277T>C *results in a phenylalanine to leucine change in *ND2*. Position 270 of *ND2 *is column 21 in Figure 5. At this position, primates have phenylalanine and other species before have leucine. *p.Phe270Leu *changes the human sequence back to the historical residue. Polyphen-2 and SIFT predict that this substitution is benign and tolerated, respectively.

**Figure 6:**
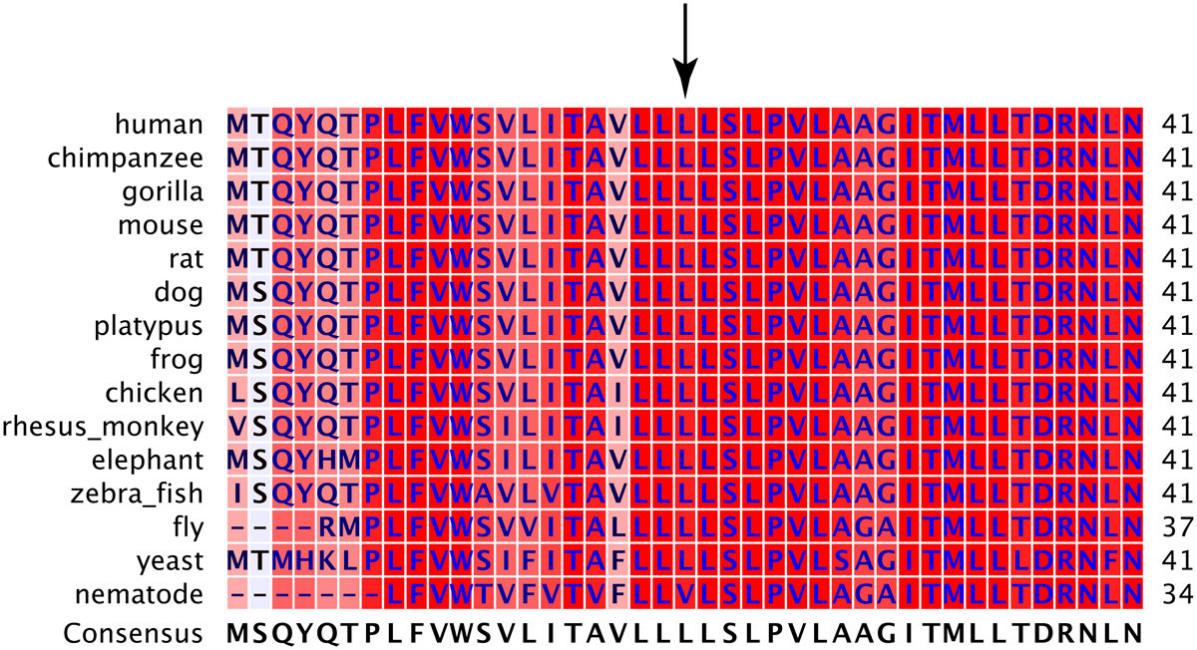
**Multiple sequence alignment of COXI**. Position 21 in the alignment corresponds to position 196 in COXI. Background colors are as described in Figure 3.

**Figure 7:**
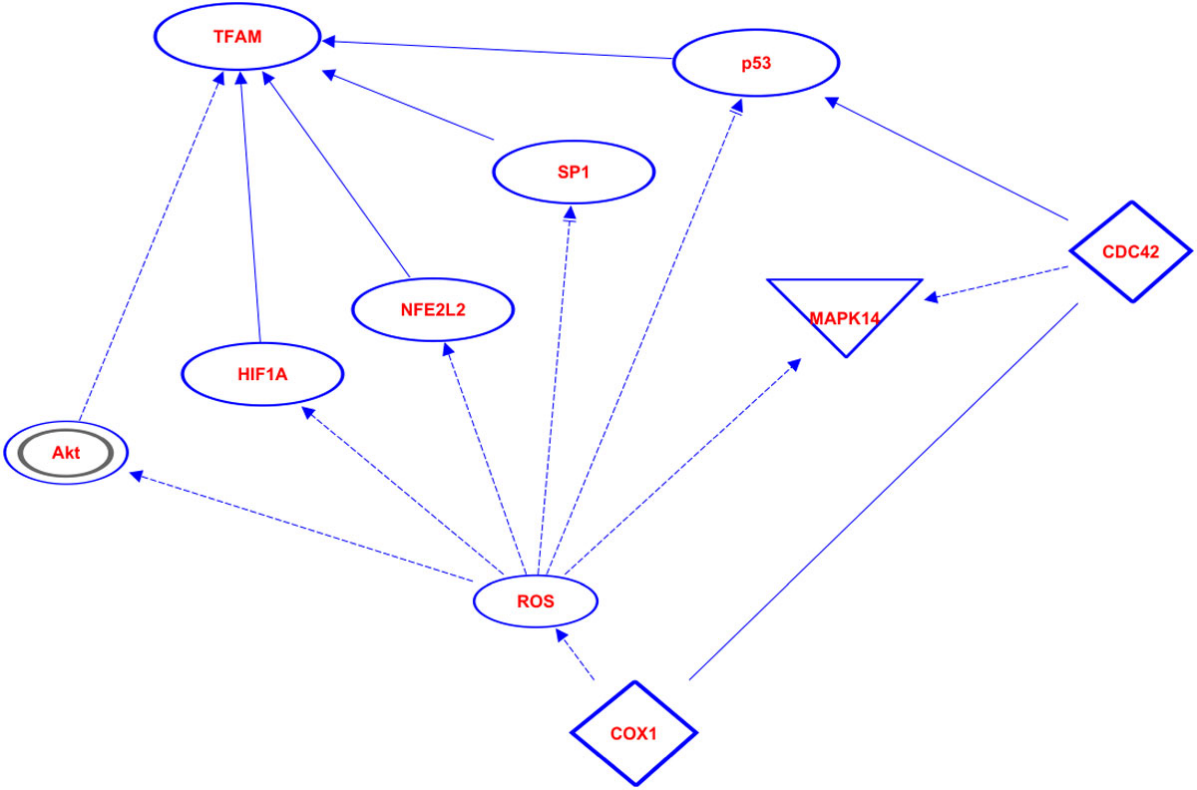
**Pathways between COXI and known regulators of mtDNA copy number**. Here we show all the known pathways between COXI and the different genes known to regulate or modify mtDNA copy number.

Next, *m.6489C>A *causes a leucine to isoleucine change at position 196 of *COXI*. This region of *COXI *is highly conserved. Position 196 is leucine in every species we examined from humans to yeast except nematodes that have valine at this position (Figure 6). Polyphen-2 predicts that this substitution is probably damaging, and SIFT also predicts that this substitution affects function, but it is a low confidence prediction. Lastly, we identified pathways in which *COXI *malfunction could cause an increase in mtDNA copy number. First we analyzed pathways for all nuclear genes known to modify mtDNA copy number and found no obvious pathways for genes other than *p53 *and *TFAM*. We identified several pathways in which *COXI *malfunction could change mtDNA copy number, the majority of which function through intermediate genes activated by reactive oxygen species (Figure 7).

## Discussion

Using 1007 full mitochondrial genome sequences we have identified sequence variation in mtDNA that affects mtDNA copy number. Two different clades were significantly associated with higher mtDNA copy number. Each of these clades represents statistically separate effects. The first was defined by branch 124 and consisted of individuals with haplogroup U5A1, and is defined by *m.9667A>G *(*p.Asn154Ser*). This variant has also been reported in D2A1, D4M1, and J1B2A haplogroups [69]; however, no individuals in our dataset belong to these haplogroups. We analyzed this substitution to determine if it likely causes *COXIII *malfunction, and then to determine whether or not it could cause the observed increase in mtDNA copy number. Our analyses suggest this substitution does not impact *COXIII *function. This conclusion is based on several lines of evidence; first, this is a high frequency, known substitution [70], second the substitution occurs in an unconserved site (Figure 3), third asparagine and glycine, two very different amino acids, appear historically in this position and a change from asparagine to the more similar serine is likely to be tolerated, and finally this position is in a short stretch of sequence located between transmembrane domains and is not a known position of importance in the heme-copper oxidase subunit III super family, of which it is a part.

While it seems likely this variant does not disrupt *COXIII *function, it is still possible that it could alter protein-protein interactions or specific dynamics associated with the electron transport chain and ultimately lead to changes in mtDNA copy number. Our initial analyses of known regulators of mtDNA copy number with *COXIII *(Figure 4) revealed no obvious mechanism for *COXIII *to directly modify mtDNA copy number; however, Pello et al [71] reported that *m.9667A>G *causes respiratory chain assembly deficiencies in patients with Leber's hereditary optic neuropathy. *TFAM *(the main known regulator of mtDNA copy number) concentration and mtDNA copy number are proportional [42]; therefore, upregulators of *TFAM *increase mtDNA copy number. *TFAM *is regulated by *NRF-1 *and *NRF-2*, and all three are sensitive to the energy needs of the cell [72,73]. Silencing of *NRF-1 *is known to lead to lower levels of *TFAM *and *NRF-1 *expression is known to increase in response to signals meant to increase energy production [73]. We propose the following model for *m.9667A>G *to increase mtDNA copy number. First, *m.9667A>G *can decrease the efficiency of complex assembly and decrease overall energy production of the mitochondria, in response, *NRF-1 *expression increases, which in turn increases *TFAM *expression, and *TFAM *expression increases mtDNA copy number.

The second clade significantly associated with higher mtDNA copy number is defined by branch 50. Branch 50 consists of eight sequence features, six of which are synonymous changes or located in intergenic regions. We focused our functional analyses on the two missense variants. The first is *m.5277T>C *(*p.Phe270Leu*) in *ND2*. Besides T2, this variant has also been reported in L1C1A1B individuals [69] and there are no L1C1A1B individuals in our dataset. This variant is in an unconserved region immediately adjacent to a low complexity region, predicted to not affect protein function, and is not novel [74,75]. These data suggest this variant is not functionally deleterious.

In contrast, there is evidence that the second of the two variants that define this clade, *m.6489C>A *(*p.Leu196Ile*), is functionally deleterious and can explain changes in mtDNA copy number. *m.6489C>A *is specific to T2 [69]. This variant in *COXI *occurs in a highly conserved region in both *COXI *and the heme-copper subunit I domain it is in, and is predicted to affect function. *COXI *and *COXII *form the catalytic center of cytochrome c oxidase (COX), or complex IV, in the respiratory complex. *m.6489C>A *is not a novel mutation and has been reported to lead to COX deficiency and a destabilization of complex IV [76]. It does appear, however, that a high threshold of mutant mtDNA is required before a phenotype appears [76,77]. The variant has only been observed in mitochondrial haplogroups T2F1 [69] and in our T2 individuals here.

*COXI *is involved in several pathways that include known regulators of mtDNA copy number (Figure 7). Mutations in mtDNA in general, and cytochrome c oxidase malfunction specifically, lead to an increase in reactive oxygen species (ROS) [78]. ROS increase expression and/or activate protein kinase B (*Akt*) [79,80], HIF-1 Alpha (*HIF1A*) [81,82], nuclear factor (erythroid-derived 2)-like 2 (*NFE2L2*) [83], *SP1 *transcription factor [84], and *p53 *[85,86]. *p53 *could also be potentially activated by *COXI *binding *CDC42 *[87,88]. Of these genes, two suppress *TFAM *(*Akt *and *HIF1A*) [89,90], and three increase *TFAM *expression (*NFE2L2, SP1*, and *p53*) [91-93].

## Conclusions

As we outlined previously, mtDNA copy number is related to several important human health phenotypes including several age-related disorders. The identification of these variants that increase mtDNA copy number has important implications in understanding the pathological processes that underlie these phenotypes. We have used bioinformatics analyses to generate hypotheses for the mechanisms by which these variants influence mtDNA copy number, successfully generating several hypotheses. Future work to characterize these mechanisms will provide important insights into the effects of mitochondrial genomic variation on mtDNA copy number and broader human phenotypes.

## List of abbreviations

mtDNA, mitochondrial genome; CNS, central nervous system, AD, Alzheimer's disease; ROS, reactive oxygen species; ETC, electron transport chain; SNV, single nucleotide variant; TFAM, mitochondrial transcription factor A; STAT3, signal transducer and activator of transcription 3; D-loop, displacement loop; COX, cytochrome C oxidase; COXI, cytochrome C oxidase 1; COXIII, cytochrome C oxidase 3; ND2, NADH dehydrogenase 2; ND5, NADH dehydrogenase 5; Akt, activate protein kinase B; HIF1A, HIF-1 Alpha; NFE2L2, nuclear factor (erythroid-derived 2)-like 2; CEPH, Centre d'Etude du Polymorphisme.

## Competing interests

The authors declare that they have no competing interests.

## Authors' contributions

PGR, TYM, RMC, and JSKK designed analyses; PGR2, TJM, and RAK performed the analyses; CDC, JTT, MCN, RGM, EO, and RAK collected the data; RMC measured mitochondrial copy number; PGR2 and SJF wrote the paper; all authors contributed to revisions and approved of the final draft.

## Supplementary Material

Additional file 1 **(docx) Mitochondrial copy number measurements**. List of mitochondrial copy number measurements, age, and gender for all individuals used in our analyses. Click here for file

Additional file 2 **(pdf) Haplotype network**. Our haplotype network was constructed using TCS and 285 full mitochondrial genomes. The arrows point to each of the three branches representing the significant contrasts. The blue arrow points to branch 124, the red to branch 121, and the green to branch 50. Click here for file

Additional file 3 **(tiff) Haplotype network**. We collapsed our haplotype network (Additional File 2) into nodes corresponding to the major mitochondrial haplogroups present in our network. Click here for file

Additional file 4 **(xlsx) Mitochondrial haplogroups**. We have listed all the major mitochondrial haplogroups as well as sub-haplogroups in our dataset. The numbers in parenthesis represent the number of individuals in our dataset that belong to the haplogroup. There were 1007 total individuals in our dataset. Click here for file

Additional file 5 **(docx) Kinship coefficients for the clade defined by branch 124**. We have listed the pairwise kinship coefficients for all the individuals in this clade. The IDs (row and column titles) correspond to the same IDs used in Additional File 1. Click here for file

Additional file 6 **(docx) Kinship coefficients for the clade defined by branch 50**. We have listed the pairwise kinship coefficients for all the individuals in this clade. The IDs (row and column titles) correspond to the same IDs used in Additional File 1. Click here for file

## Declarations

This work was supported by grants from NIH (R01AG11380, R01AG21136, R01AG31272, R01AG042611), the Alzheimer's Association (MNIRG-11-205368) and the Utah Science, Technology, and Research initiative (USTAR), and the Brigham Young University Gerontology Program (JSKK).

This article has been published as part of *BMC Bioinformatics *Volume 15 Supplement 7, 2014: Selected articles from the 10th Annual Biotechnology and Bioinformatics Symposium (BIOT 2013). The full contents of the supplement are available online at http://www.biomedcentral.com/bmcbioinformatics/supplements/15/S7
