# Supplementary material for: Mitochondrial genomic variation associated with higher mitochondrial copy number: the Cache County Study on Memory Health and Aging
Source: BMC Bioinformatics. 2014 May 28;15(Suppl 7):S6. doi: 10.1186/1471-2105-15-S7-S6 (PMC4110732; doi:10.1186/1471-2105-15-S7-S6)
Supplement: Additional file 1 — (docx) Mitochondrial copy number measurements. List of mitochondrial copy number measurements, age, and gender for all individuals used in our analyses. [file 1471-2105-15-S7-S6-S1.docx]

| **Cache ID** | **Mitochondria #** | **Age** | **Gender** |
| --- | --- | --- | --- |
| 4 | 2.8 | 75.28474428 | female |
| 6 | 1.84 | 76.87000329 | male |
| 9 | 1.99 | 75.69543314 | female |
| 10 | 2.48 | 68.92180484 | male |
| 11 | 5.18 | 68.77669478 | female |
| 16 | 2.58 | 69.16821816 | male |
| 17 | N/A | 78.0308838 | female |
| 18 | N/A | 74.5427664 | male |
| 19 | 2.61 | 74.14576717 | female |
| 26 | 1.86 | 70.63300843 | male |
| 30 | 3.36 | 88.6540357 | female |
| 31 | 2.15 | 68.93001862 | male |
| 32 | 2.72 | 70.19494031 | female |
| 34 | 3.21 | 86.909977 | female |
| 37 | 2.23 | 78.56477932 | male |
| 41 | 3.23 | 77.06713394 | female |
| 45 | 2.34 | 67.61033841 | female |
| 54 | 2.93 | 76.70025189 | male |
| 55 | N/A | 78.12944913 | male |
| 59 | 4.4 | 77.35461614 | female |
| 65 | 1.59 | 88.31727084 | female |
| 66 | N/A | 83.63541781 | female |
| 67 | N/A | 77.08082357 | female |
| 72 | 3.48 | 91.74515387 | male |
| 73 | N/A | 90.05585369 | female |
| 78 | 1.36 | 74.58109736 | male |
| 86 | 1.43 | 73.05333479 | female |
| 87 | 2.51 | 78.30467638 | male |
| 100 | 3.71 | 85.73266893 | female |
| 101 | 3.32 | 71.00262841 | male |
| 125 | 2.45 | 84.8154638 | female |
| 130 | 4.79 | 67.23524258 | female |
| 131 | 3.19 | 90.50761143 | male |
| 137 | 2.57 | 69.45296244 | male |
| 138 | 2.11 | 81.03165042 | male |
| 140 | 4.84 | 81.36841529 | female |
| 142 | N/A | 72.43182565 | female |
| 144 | 2.58 | 79.05486803 | male |
| 147 | 2.08 | 65.97579674 | female |
| 162 | 1.95 | 87.50410689 | female |
| 167 | 2.26 | 67.24619428 | female |
| 168 | 3.91 | 88.01062315 | female |
| 176 | 3.09 | 71.30653817 | female |
| 185 | 1.61 | 66.71503669 | male |
| 189 | 2.45 | 84.58274012 | female |
| 194 | 3.38 | 66.82181579 | female |
| 195 | 2.76 | 69.12167342 | female |
| 197 | 1.17 | 80.12813492 | female |
| 200 | N/A | 68.31398532 | male |
| 202 | 4.11 | 71.55568941 | male |
| 203 | 2.68 | 75.49008871 | male |
| 225 | N/A | 85.63957945 | female |
| 230 | N/A | 87.32614172 | female |
| 232 | 1.83 | 77.07808564 | female |
| 237 | 3.64 | 73.27510678 | female |
| 244 | 2.17 | 81.66137334 | male |
| 252 | 2.61 | 94.01215639 | female |
| 264 | 2.73 | 74.21147738 | male |
| 275 | 2.56 | 69.93483737 | female |
| 279 | 3.27 | 75.10404118 | female |
| 280 | 2.97 | 73.20939656 | male |
| 289 | 3.61 | 72.93286606 | male |
| 294 | 2.63 | 86.28572993 | male |
| 295 | 2.46 | 70.89311138 | female |
| 298 | 2.47 | 67.76092432 | male |
| 309 | 2.09 | 67.71985544 | female |
| 316 | 3.78 | 69.948527 | female |
| 333 | 3.03 | 87.86277516 | female |
| 339 | N/A | 81.305443 | female |
| 346 | 2.73 | 99.15124302 | female |
| 348 | 3.41 | 84.59095389 | male |
| 358 | 3.47 | 77.8501807 | female |
| 361 | N/A | 89.79848866 | female |
| 365 | 2.87 | 66.54528529 | female |
| 366 | 2.34 | 68.91359106 | male |
| 367 | 2.23 | 72.08410908 | female |
| 374 | 3.06 | 74.56466981 | male |
| 375 | 3.89 | 70.44682948 | female |
| 382 | 2.99 | 69.51867265 | male |
| 384 | 2.57 | 74.13207754 | female |
| 389 | 1.5 | 69.12714927 | male |
| 390 | 3.56 | 67.22976673 | male |
| 393 | 3.22 | 71.13131092 | female |
| 400 | 3.23 | 73.11083123 | female |
| 405 | 1.95 | 73.27236885 | male |
| 408 | 3.5 | 65.61986639 | female |
| 412 | 2.22 | 70.73431169 | female |
| 417 | 2.09 | 87.24126602 | female |
| 426 | 2.08 | 71.79388895 | male |
| 431 | 2.94 | 67.77735188 | male |
| 443 | 3.31 | 73.92399518 | female |
| 446 | 0.94 | 88.77176651 | female |
| 454 | N/A | 71.19154529 | male |
| 455 | 3.69 | 70.5864637 | female |
| 460 | 3.93 | 90.06406746 | female |
| 465 | 3.19 | 67.20786332 | male |
| 466 | 2.93 | 84.45131968 | male |
| 469 | 1.79 | 74.75632461 | male |
| 470 | 1.93 | 70.55908444 | male |
| 471 | 0.99 | 66.75610557 | male |
| 472 | 2.82 | 86.90450115 | female |
| 477 | 2.51 | 71.11488336 | male |
| 490 | N/A | 80.51692038 | male |
| 510 | 2.88 | 75.59960574 | female |
| 515 | N/A | 68.77669478 | female |
| 516 | 4.1 | 70.17851276 | male |
| 525 | 2.4 | 91.93133282 | female |
| 529 | 2.77 | 72.28671558 | male |
| 531 | 2.17 | 69.0340598 | female |
| 537 | 2.35 | 78.73453072 | female |
| 541 | 1.48 | 72.57967364 | female |
| 543 | 2.88 | 67.56105574 | male |
| 548 | 3.1 | 71.10666959 | female |
| 550 | 3.17 | 74.41134596 | male |
| 552 | 2.72 | 89.94633666 | male |
| 557 | 1.94 | 71.60223415 | female |
| 559 | 2.36 | 85.76826196 | male |
| 560 | 3.33 | 83.00295696 | female |
| 561 | 2.32 | 75.16153762 | male |
| 566 | 3.25 | 67.23250465 | male |
| 567 | 2.45 | 77.42306429 | male |
| 568 | N/A | 76.44014894 | female |
| 582 | 4.3 | 81.76541452 | female |
| 587 | 2.96 | 80.62917534 | male |
| 590 | 4.05 | 76.69203811 | female |
| 592 | 2.15 | 75.26831672 | female |
| 596 | 2.33 | 72.46741868 | male |
| 598 | 2.2 | 68.07030993 | female |
| 607 | N/A | 71.0656007 | female |
| 611 | N/A | 75.56948856 | female |
| 624 | 3.8 | 82.2199102 | female |
| 625 | 3.07 | 89.31113788 | female |
| 630 | 2.3 | 78.06100099 | male |
| 633 | 1.82 | 70.39480889 | female |
| 640 | 2.44 | 82.88248823 | female |
| 645 | N/A | 75.06023437 | male |
| 646 | 2.05 | 71.70627533 | male |
| 649 | 2.52 | 68.40433687 | male |
| 652 | 3.12 | 67.9607929 | male |
| 653 | N/A | 96.6925857 | female |
| 655 | N/A | 88.22965721 | male |
| 657 | 2.83 | 86.33227467 | male |
| 658 | 1.96 | 84.69225715 | female |
| 660 | 3.33 | 77.98433906 | male |
| 665 | 3.98 | 72.54134268 | female |
| 668 | 2.04 | 90.4610667 | male |
| 675 | 3.08 | 86.92640456 | male |
| 684 | N/A | 72.44551528 | female |
| 685 | 2.35 | 76.77417588 | female |
| 688 | 1.46 | 74.39218048 | female |
| 689 | 3.24 | 75.59412989 | male |
| 705 | 3.66 | 75.54484722 | male |
| 709 | 2.24 | 67.74723469 | female |
| 711 | 3.99 | 96.09571788 | male |
| 732 | 2.39 | 65.89913482 | male |
| 744 | N/A | 68.95739788 | male |
| 752 | 3.12 | 65.81973497 | male |
| 753 | 3 | 89.66706823 | male |
| 755 | 2.45 | 72.49753587 | male |
| 759 | 1.57 | 76.46479027 | male |
| 771 | 2.92 | 84.11181689 | female |
| 774 | 1.05 | 78.32931771 | female |
| 777 | 2.27 | 75.63519877 | female |
| 792 | N/A | 74.49895959 | female |
| 807 | 2.25 | 77.86660826 | male |
| 813 | 3.42 | 74.45241485 | female |
| 822 | 3.46 | 82.23633775 | male |
| 832 | 6.12 | 66.46040959 | male |
| 837 | 3.86 | 74.73168328 | male |
| 841 | 3.38 | 90.94294163 | female |
| 847 | 1.54 | 75.4462819 | male |
| 853 | 3.31 | 68.88894973 | female |
| 858 | N/A | 90.26667397 | male |
| 868 | 1.24 | 66.91216734 | female |
| 869 | 2.43 | 67.37213887 | female |
| 872 | 1.98 | 67.21881503 | female |
| 873 | N/A | 82.56488884 | female |
| 874 | 2.48 | 81.17676049 | female |
| 875 | 3.29 | 90.50213558 | male |
| 879 | N/A | 68.89990143 | female |
| 892 | N/A | 65.4829701 | female |
| 894 | 2.45 | 86.14609572 | female |
| 904 | 2.55 | 85.68064834 | female |
| 915 | 3.44 | 66.87931223 | male |
| 917 | 2.16 | 70.01971307 | male |
| 919 | N/A | 67.46249042 | male |
| 925 | 2.86 | 77.26974044 | female |
| 938 | 2.45 | 82.38144782 | female |
| 950 | 3.33 | 70.39754682 | male |
| 960 | 4.06 | 70.42218815 | female |
| 971 | N/A | 74.60026284 | male |
| 973 | 3.07 | 72.02661264 | female |
| 988 | 0.68 | 67.16679444 | male |
| 997 | 2.34 | 71.92530939 | male |
| 998 | N/A | 69.44474866 | female |
| 1000 | 2.46 | 68.42624028 | female |
| 1002 | 2.66 | 69.91840981 | female |
| 1005 | 2.92 | 70.72883583 | female |
| 1016 | 2.27 | 68.41255065 | female |
| 1017 | 2.02 | 66.09626547 | female |
| 1032 | 2.1 | 75.18891688 | female |
| 1053 | 1.73 | 69.81710656 | male |
| 1054 | 1.77 | 68.60694338 | female |
| 1064 | 1.81 | 69.82258241 | female |
| 1069 | N/A | 65.5678458 | female |
| 1074 | 4 | 81.89135911 | male |
| 1079 | 2 | 90.5568941 | female |
| 1089 | N/A | 68.28386814 | female |
| 1095 | N/A | 65.42821159 | female |
| 1119 | 1.98 | 88.03526448 | female |
| 1120 | 2.12 | 71.08476618 | female |
| 1121 | 2.54 | 67.71164166 | female |
| 1123 | 2.75 | 71.77198554 | female |
| 1162 | 1.46 | 77.61471909 | male |
| 1164 | N/A | 70.99989048 | male |
| 1165 | 1.39 | 72.39349469 | female |
| 1167 | 2.19 | 72.63990801 | female |
| 1188 | 1.74 | 67.1421531 | female |
| 1194 | N/A | 79.86803198 | male |
| 1205 | 2.1 | 72.47015661 | female |
| 1213 | 2.54 | 67.12298762 | male |
| 1223 | 3.21 | 73.80078852 | male |
| 1227 | N/A | 67.36392509 | male |
| 1232 | 2.45 | 66.35910634 | female |
| 1234 | 0.81 | 71.2353521 | male |
| 1235 | 3.53 | 70.24969883 | male |
| 1245 | 3.15 | 70.13744387 | female |
| 1248 | 2.68 | 69.66104479 | female |
| 1257 | N/A | 71.46259993 | male |
| 1259 | 0.76 | 87.48220348 | female |
| 1264 | 2.41 | 79.63530829 | female |
| 1265 | 3.4 | 68.34684043 | male |
| 1288 | 1.41 | 72.19362611 | male |
| 1294 | 2.93 | 68.10042712 | male |
| 1301 | N/A | 77.38473333 | male |
| 1303 | 1.56 | 72.54134268 | female |
| 1308 | 2.78 | 70.77264265 | male |
| 1309 | 3.5 | 82.08301391 | male |
| 1322 | 3.71 | 77.19581645 | female |
| 1333 | 2.01 | 82.16515168 | male |
| 1335 | 2.12 | 71.27094513 | male |
| 1336 | 2.8 | 71.07929033 | female |
| 1339 | 2.85 | 72.28397766 | female |
| 1342 | 1.52 | 66.64932647 | female |
| 1351 | 1.63 | 70.49885007 | male |
| 1358 | 1.94 | 78.08564232 | male |
| 1368 | 3.53 | 77.55722265 | male |
| 1373 | 2.57 | 95.87120797 | female |
| 1381 | 2.4 | 81.83933852 | female |
| 1383 | 3.5 | 71.74460629 | female |
| 1388 | N/A | 73.69127149 | female |
| 1392 | 1.4 | 89.39327565 | male |
| 1395 | 2.4 | 82.9755777 | female |
| 1398 | 2.45 | 71.49819297 | female |
| 1415 | N/A | 92.79925528 | female |
| 1429 | N/A | 75.22998576 | female |
| 1430 | 1.99 | 68.58230205 | female |
| 1436 | N/A | 82.30204797 | female |
| 1439 | 2.85 | 75.71186069 | female |
| 1442 | 2.01 | 71.58306867 | female |
| 1459 | 1.61 | 68.21268207 | female |
| 1470 | 2.87 | 72.71656993 | female |
| 1478 | 3.54 | 83.21377724 | female |
| 1479 | 3.46 | 82.94819844 | female |
| 1489 | N/A | 72.49479794 | female |
| 1497 | N/A | 70.63027051 | female |
| 1498 | 1.03 | 73.09987953 | male |
| 1502 | 1.03 | 71.65425474 | male |
| 1510 | 3.15 | 68.57956412 | female |
| 1539 | N/A | 86.01467528 | female |
| 1541 | 2.15 | 72.08137115 | female |
| 1555 | N/A | 80.55798927 | female |
| 1559 | 3.08 | 79.91183879 | male |
| 1575 | 3.37 | 87.14817654 | female |
| 1578 | 2.95 | 76.97678239 | male |
| 1589 | 2.31 | 65.93472785 | female |
| 1590 | N/A | 69.61723798 | male |
| 1601 | 2.67 | 70.40849852 | male |
| 1602 | 2.25 | 68.92180484 | female |
| 1610 | 2.28 | 71.80757858 | female |
| 1627 | 2.57 | 83.591611 | male |
| 1630 | 3.67 | 75.68448144 | female |
| 1634 | 2.05 | 67.28452524 | female |
| 1653 | 3.85 | 81.23699485 | female |
| 1655 | 3.14 | 70.17577483 | male |
| 1656 | 2.67 | 90.66914905 | female |
| 1665 | 2.42 | 89.51374439 | female |
| 1678 | 3.08 | 74.67966269 | male |
| 1695 | 1.97 | 66.67670573 | female |
| 1701 | 1.73 | 66.03329318 | female |
| 1708 | 2.32 | 77.64483627 | female |
| 1715 | 3.5 | 73.69400942 | male |
| 1716 | 3.4 | 73.94042274 | female |
| 1725 | 2.13 | 70.55634651 | male |
| 1727 | 3.33 | 84.35549228 | female |
| 1736 | N/A | 69.80889278 | male |
| 1745 | 2.19 | 81.88588325 | male |
| 1749 | 2.13 | 69.46665206 | female |
| 1750 | 2.21 | 71.15869018 | male |
| 1751 | 1.73 | 77.55996057 | male |
| 1764 | 2.67 | 85.40411784 | female |
| 1772 | 3.8 | 82.55941299 | male |
| 1778 | N/A | 72.13339174 | female |
| 1781 | N/A | 74.95071734 | female |
| 1785 | 1.57 | 77.88851166 | male |
| 1787 | 2.03 | 70.6083671 | male |
| 1792 | 1.52 | 67.79651736 | male |
| 1796 | 3.39 | 67.06275326 | male |
| 1797 | 2.31 | 71.94721279 | female |
| 1825 | N/A | 81.53269083 | female |
| 1835 | N/A | 81.58471142 | male |
| 1840 | 1.95 | 78.66334465 | female |
| 1855 | 3.53 | 79.80232176 | female |
| 1871 | 2.33 | 70.7041945 | male |
| 1881 | 2.49 | 71.94721279 | female |
| 1887 | 3.1 | 71.15869018 | female |
| 1893 | 2.49 | 81.40400832 | female |
| 1907 | N/A | 71.8732888 | male |
| 1910 | 3.73 | 89.96824006 | male |
| 1930 | 2.44 | 72.98214872 | male |
| 1948 | 2.9 | 80.09527982 | male |
| 1955 | 3.28 | 88.27346402 | female |
| 1968 | N/A | 66.04972073 | female |
| 1976 | 3.6 | 86.72106012 | female |
| 1977 | N/A | 93.83692914 | female |
| 1992 | 3.72 | 85.40685577 | male |
| 1993 | 1.77 | 78.6797722 | female |
| 2029 | 2.49 | 67.18322199 | male |
| 2034 | 2.71 | 70.68502902 | female |
| 2036 | 2.46 | 79.0411784 | female |
| 2058 | 2.24 | 74.03351221 | male |
| 2060 | N/A | 66.44398204 | female |
| 2065 | 2.46 | 91.80812616 | male |
| 2068 | 3.02 | 66.93407075 | female |
| 2077 | N/A | 83.12342569 | female |
| 2083 | 2.3 | 78.78107546 | female |
| 2086 | 0.98 | 86.78403242 | female |
| 2112 | 3.21 | 66.6849195 | male |
| 2137 | 1.12 | 74.86036579 | female |
| 2146 | 2.26 | 75.29295805 | female |
| 2150 | N/A | 69.93483737 | female |
| 2152 | 3.03 | 70.37016756 | male |
| 2159 | 2.97 | 81.50257365 | female |
| 2171 | 4.19 | 69.45296244 | female |
| 2175 | 2.71 | 79.34235024 | male |
| 2178 | 3.42 | 73.56532691 | male |
| 2179 | N/A | 71.63782718 | female |
| 2182 | 3.11 | 97.42087395 | female |
| 2189 | N/A | 81.11105027 | male |
| 2195 | N/A | 67.6842624 | female |
| 2200 | 2.75 | 82.79213668 | female |
| 2201 | 3.57 | 74.22790494 | female |
| 2202 | N/A | 69.75413427 | female |
| 2205 | 2.01 | 79.5832877 | male |
| 2206 | N/A | 93.11685467 | female |
| 2224 | 0.94 | 73.05333479 | male |
| 2229 | 4.23 | 65.52403899 | male |
| 2230 | 3.93 | 72.21552951 | male |
| 2231 | 2.46 | 66.64385062 | male |
| 2246 | 4.77 | 75.95827401 | female |
| 2257 | 2.16 | 65.58153543 | female |
| 2284 | 1.82 | 68.33041288 | female |
| 2285 | 1.51 | 70.69871865 | female |
| 2295 | N/A | 88.47880845 | male |
| 2298 | 1.3 | 84.17478918 | female |
| 2301 | 2.86 | 71.40236557 | female |
| 2312 | 3.53 | 68.70824663 | male |
| 2318 | 3.5 | 69.90198226 | female |
| 2324 | N/A | 69.28594897 | male |
| 2345 | 4.08 | 66.15376191 | male |
| 2346 | 2.95 | 78.2225386 | female |
| 2347 | 2.17 | 86.54035703 | male |
| 2370 | 1.59 | 71.84043369 | male |
| 2373 | N/A | 66.05793451 | female |
| 2377 | 3.52 | 87.73135473 | male |
| 2386 | 2.64 | 81.74077319 | female |
| 2391 | 1.22 | 65.5568941 | female |
| 2392 | 2.65 | 68.1195926 | male |
| 2393 | 3.6 | 94.62545176 | female |
| 2417 | 2.56 | 65.93198992 | female |
| 2420 | 3.19 | 86.09955098 | female |
| 2428 | N/A | 70.39754682 | male |
| 2439 | 3.07 | 70.21410579 | female |
| 2442 | 1.44 | 73.41747892 | female |
| 2449 | 1.91 | 67.16131859 | female |
| 2466 | 2.7 | 78.87690286 | female |
| 2470 | 2.31 | 70.44409156 | male |
| 2478 | N/A | 82.0501588 | male |
| 2484 | 2.9 | 77.7434016 | male |
| 2493 | 1.58 | 65.81973497 | male |
| 2494 | 3.57 | 69.31606615 | male |
| 2507 | 2.23 | 65.72664549 | female |
| 2521 | N/A | 65.61439054 | female |
| 2522 | 1.61 | 71.20523491 | female |
| 2528 | 3.53 | 73.9897054 | male |
| 2530 | 3.36 | 77.34092651 | male |
| 2532 | N/A | 77.1054649 | female |
| 2540 | N/A | 89.03186946 | female |
| 2558 | 5.34 | 74.35658745 | female |
| 2560 | 2.53 | 80.14730041 | female |
| 2576 | 2.15 | 80.93856095 | male |
| 2579 | 2.32 | 90.90187274 | male |
| 2580 | 2.53 | 66.18387909 | male |
| 2584 | 2.87 | 88.87580769 | male |
| 2588 | 3.52 | 71.24904173 | female |
| 2597 | 3.38 | 65.84985215 | female |
| 2600 | 2.95 | 70.02792684 | female |
| 2611 | 0.98 | 66.95871208 | female |
| 2612 | 1.86 | 70.48242252 | male |
| 2618 | 2.12 | 67.42142153 | male |
| 2622 | 2.4 | 66.77253313 | male |
| 2623 | 2.16 | 70.36742964 | male |
| 2624 | N/A | 79.03570255 | male |
| 2626 | N/A | 77.30807141 | male |
| 2627 | 2.82 | 69.98685796 | female |
| 2628 | 1.79 | 77.06987187 | male |
| 2629 | 3.15 | 67.41046983 | male |
| 2634 | 2.87 | 93.00186179 | female |
| 2636 | N/A | 70.62205673 | male |
| 2640 | N/A | 74.35658745 | female |
| 2645 | N/A | 68.42076443 | female |
| 2654 | N/A | 71.60223415 | female |
| 2660 | 2.71 | 73.89935385 | female |
| 2661 | N/A | 72.65359763 | male |
| 2663 | 2.42 | 67.28178732 | female |
| 2684 | 2.12 | 68.10590297 | female |
| 2686 | 3.19 | 87.04413536 | female |
| 2687 | 3.23 | 78.03362173 | male |
| 2694 | 3.39 | 90.35976344 | female |
| 2700 | 1.72 | 69.30511445 | male |
| 2701 | N/A | 82.76201949 | male |
| 2702 | 2.06 | 66.15376191 | female |
| 2710 | N/A | 68.71646041 | female |
| 2713 | 3.08 | 80.45121016 | male |
| 2720 | 1.85 | 82.48822692 | male |
| 2722 | 2.06 | 71.1395247 | male |
| 2723 | 4.17 | 71.49545504 | female |
| 2727 | 1.71 | 76.07052897 | male |
| 2731 | 4.98 | 74.75358668 | female |
| 2735 | 3.49 | 76.37991458 | male |
| 2752 | 4.24 | 82.38144782 | male |
| 2754 | 1.38 | 66.22768591 | male |
| 2758 | 2.59 | 89.51100646 | male |
| 2759 | 1.19 | 69.93483737 | female |
| 2765 | 4.35 | 74.87405542 | male |
| 2775 | 2.93 | 76.34705947 | female |
| 2791 | 3.65 | 72.79323185 | female |
| 2800 | 3.54 | 82.62238528 | female |
| 2806 | N/A | 73.38736173 | female |
| 2817 | 2.73 | 75.30938561 | male |
| 2827 | N/A | 69.19833534 | male |
| 2830 | 2.09 | 71.01358011 | female |
| 2837 | 1.72 | 73.80626437 | female |
| 2842 | 4.34 | 80.0186179 | female |
| 2853 | N/A | 69.87186508 | male |
| 2864 | 0.91 | 68.78490855 | female |
| 2868 | 1.38 | 79.52579126 | male |
| 2885 | 1.37 | 68.09221334 | female |
| 2898 | 2.58 | 92.29547695 | male |
| 2910 | 3.08 | 77.05618224 | female |
| 2916 | 2.33 | 65.60891469 | male |
| 2930 | 1.24 | 68.74110174 | female |
| 2939 | 3.19 | 67.24345636 | male |
| 2952 | N/A | 76.52502464 | female |
| 2953 | 3.32 | 72.17993648 | female |
| 2957 | N/A | 78.85773738 | male |
| 2961 | N/A | 74.46336655 | female |
| 2966 | 2.83 | 90.81152119 | female |
| 2973 | N/A | 81.37389114 | female |
| 2976 | 2.22 | 66.89573979 | male |
| 2987 | 3.97 | 67.82115869 | female |
| 2989 | 2.2 | 91.22494798 | female |
| 2995 | N/A | 65.98948637 | female |
| 3001 | 1.87 | 77.62293287 | female |
| 3006 | 4.32 | 84.41298872 | male |
| 3010 | 2 | 75.96101194 | male |
| 3015 | N/A | 71.54199978 | female |
| 3021 | 1.84 | 75.2655788 | female |
| 3036 | 3.07 | 69.82258241 | male |
| 3037 | 1.96 | 71.09297996 | female |
| 3038 | 2.33 | 72.4263498 | male |
| 3056 | 3.94 | 76.41276969 | male |
| 3060 | 0.87 | 72.74668711 | female |
| 3074 | 2.21 | 72.13886759 | female |
| 3077 | N/A | 78.07742854 | male |
| 3084 | 1.48 | 77.37651955 | female |
| 3096 | 1.78 | 70.84930457 | female |
| 3097 | 3.36 | 67.31738035 | female |
| 3099 | 1.88 | 69.26678348 | male |
| 3104 | 2.34 | 70.73157376 | female |
| 3108 | 2.81 | 86.27751615 | female |
| 3113 | 3.57 | 79.019275 | male |
| 3115 | 3.72 | 84.54440916 | female |
| 3123 | N/A | 72.85346621 | male |
| 3124 | 2.51 | 67.07918081 | male |
| 3144 | 3.35 | 78.24717994 | female |
| 3145 | 1.88 | 70.48516044 | male |
| 3155 | 4.71 | 68.3112474 | male |
| 3161 | 1.17 | 73.77614719 | female |
| 3162 | 2.93 | 92.48713175 | male |
| 3187 | 3.65 | 73.83364363 | female |
| 3188 | 1.6 | 82.06658635 | male |
| 3191 | 3.13 | 80.38823787 | male |
| 3192 | 1.18 | 71.57485489 | male |
| 3199 | 2.91 | 72.82061111 | male |
| 3202 | 2.7 | 83.03854999 | male |
| 3211 | N/A | 82.38966159 | female |
| 3226 | 5.18 | 75.06844814 | female |
| 3238 | 3.58 | 89.30018618 | female |
| 3243 | N/A | 65.82521082 | female |
| 3263 | 1.53 | 80.70857518 | male |
| 3272 | 2.64 | 69.34618333 | female |
| 3276 | 3.07 | 70.90680101 | female |
| 3292 | 2.66 | 75.88708794 | female |
| 3294 | 0.92 | 75.49282663 | female |
| 3295 | 3.91 | 81.18771219 | female |
| 3299 | 2.67 | 80.71952689 | male |
| 3306 | N/A | 91.3864856 | female |
| 3311 | 1.95 | 84.13645822 | female |
| 3312 | 4.97 | 87.25769357 | female |
| 3326 | 2.99 | 67.51451101 | female |
| 3328 | 2.09 | 65.37892892 | female |
| 3329 | 1.5 | 68.0566203 | male |
| 3332 | 1.49 | 78.39502793 | female |
| 3333 | 3.29 | 79.46281897 | male |
| 3339 | 3.7 | 75.83506735 | male |
| 3343 | 2.55 | 67.01894645 | male |
| 3372 | N/A | 70.24969883 | male |
| 3374 | 3.05 | 88.76629066 | female |
| 3378 | 0.94 | 82.15146205 | female |
| 3380 | 1.93 | 81.5080495 | female |
| 3385 | 2.71 | 82.4225167 | male |
| 3393 | 4.2 | 74.10469828 | male |
| 3398 | 2.37 | 79.86529405 | female |
| 3402 | N/A | 72.97667287 | male |
| 3433 | N/A | 87.68480999 | male |
| 3436 | N/A | 75.27105465 | male |
| 3438 | 3.12 | 73.7980506 | male |
| 3440 | 3.63 | 85.19603548 | female |
| 3453 | 2.76 | 72.24838462 | female |
| 3455 | 1.74 | 68.65622604 | male |
| 3465 | 1.87 | 78.28824882 | female |
| 3469 | 4.43 | 95.09911291 | female |
| 3490 | 3.2 | 79.85708028 | female |
| 3491 | N/A | 77.97338736 | female |
| 3514 | N/A | 81.95706932 | female |
| 3518 | 1.51 | 65.94294163 | female |
| 3524 | 2.72 | 87.81896835 | male |
| 3537 | 1.49 | 88.76081481 | male |
| 3539 | 2.49 | 71.38867594 | male |
| 3557 | N/A | 73.63651298 | female |
| 3595 | 2.75 | 71.35582083 | female |
| 3602 | 2.41 | 70.12649217 | male |
| 3604 | 2.37 | 75.60781952 | female |
| 3607 | 4.88 | 74.33468404 | female |
| 3613 | 2.59 | 68.09495126 | female |
| 3644 | 4.56 | 66.25506516 | female |
| 3645 | N/A | 69.50772095 | female |
| 3646 | 2.01 | 70.18946446 | female |
| 3656 | 2.02 | 67.99091009 | female |
| 3661 | N/A | 75.97470157 | male |
| 3685 | 2.94 | 76.59347279 | female |
| 3692 | 3.21 | 75.02464133 | male |
| 3694 | N/A | 72.99583835 | male |
| 3695 | 3.36 | 76.62085204 | female |
| 3701 | 1.76 | 77.69411894 | female |
| 3706 | 2.19 | 93.39064725 | male |
| 3710 | 1.85 | 83.5286387 | male |
| 3742 | 3.76 | 67.47618005 | female |
| 3751 | N/A | 74.88500712 | male |
| 3754 | 4.21 | 73.05881065 | female |
| 3755 | 3.06 | 81.1356916 | female |
| 3758 | 2.63 | 88.90044902 | female |
| 3760 | 1.12 | 66.96692586 | female |
| 3789 | 3.58 | 85.82849633 | male |
| 3792 | 2.84 | 77.13010623 | female |
| 3797 | 3.47 | 67.70616581 | female |
| 3802 | 2.55 | 70.21410579 | male |
| 3819 | 1.56 | 65.67736283 | female |
| 3822 | 3.44 | 69.99780966 | male |
| 3823 | 3.13 | 66.75062972 | male |
| 3825 | N/A | 80.68393385 | male |
| 3859 | 3.77 | 73.76519549 | female |
| 3860 | 2.63 | 90.78140401 | male |
| 3871 | 3.21 | 93.74931552 | female |
| 3878 | 2.41 | 72.63169423 | male |
| 3880 | 3.34 | 72.57693571 | male |
| 3883 | 3.84 | 85.86956522 | female |
| 3903 | 3.02 | 71.64877889 | female |
| 3912 | 2.37 | 66.16471361 | female |
| 3916 | N/A | 66.5671887 | female |
| 3917 | N/A | 67.99638594 | male |
| 3918 | 4.07 | 86.91271493 | female |
| 3922 | N/A | 81.46698062 | female |
| 3931 | 3.28 | 73.1710656 | female |
| 3932 | 1.9 | 74.01708466 | male |
| 3938 | N/A | 74.71525572 | male |
| 3940 | 2.43 | 68.41528858 | male |
| 3942 | 2.89 | 72.23743292 | female |
| 3946 | 2.1 | 85.8996824 | female |
| 3953 | 3.91 | 81.0398642 | female |
| 3982 | 1.4 | 88.21870551 | male |
| 3987 | 2.23 | 75.59412989 | female |
| 3995 | N/A | 72.52765305 | male |
| 3999 | N/A | 66.09352754 | male |
| 4004 | 2.58 | 69.49403132 | female |
| 4007 | N/A | 83.32055635 | female |
| 4018 | 2.99 | 76.97404446 | female |
| 4030 | 4.29 | 82.41704085 | male |
| 4037 | 1.24 | 76.26218377 | male |
| 4046 | 2.71 | 87.15912824 | female |
| 4047 | 3.3 | 69.61450005 | male |
| 4049 | 2.84 | 70.67955317 | male |
| 4059 | 2.15 | 70.48789837 | female |
| 4061 | 1.15 | 78.55930347 | male |
| 4064 | 2.31 | 80.61822363 | male |
| 4066 | 1.9 | 81.62304238 | female |
| 4067 | 2.46 | 80.74690614 | male |
| 4074 | 3.21 | 75.67900559 | female |
| 4076 | 1.94 | 70.25517468 | female |
| 4094 | N/A | 78.26360749 | male |
| 4102 | N/A | 66.74515387 | female |
| 4104 | 4.06 | 67.13667725 | female |
| 4114 | 1.6 | 69.10798379 | male |
| 4116 | 3.28 | 69.39546599 | male |
| 4123 | 1.84 | 70.8739459 | male |
| 4130 | 3.26 | 75.63519877 | male |
| 4132 | 2.96 | 68.78764648 | female |
| 4136 | 2.25 | 70.23053335 | female |
| 4137 | 2.36 | 73.82816778 | male |
| 4150 | 3.19 | 66.94228453 | male |
| 4157 | 2.79 | 79.81053554 | male |
| 4161 | 4.38 | 80.57715475 | female |
| 4162 | 2.36 | 76.08695652 | female |
| 4163 | 4.09 | 70.30719527 | male |
| 4165 | 3.57 | 70.73157376 | female |
| 4169 | 4.47 | 87.12901106 | male |
| 4174 | 1.74 | 77.85565655 | male |
| 4193 | 2.52 | 86.50750192 | female |
| 4209 | 2.92 | 87.52601029 | male |
| 4210 | 2.35 | 68.07030993 | male |
| 4213 | 2.99 | 65.89913482 | female |
| 4269 | 2.56 | 77.97886321 | female |
| 4283 | N/A | 88.85390428 | female |
| 4284 | 2.7 | 68.37695762 | female |
| 4289 | N/A | 76.25123207 | female |
| 4293 | 3.29 | 66.60004381 | male |
| 4299 | N/A | 76.42372139 | female |
| 4304 | 2.31 | 71.17237981 | female |
| 4313 | 1.8 | 80.07611434 | male |
| 4322 | 3.28 | 70.1292301 | female |
| 4325 | 3.99 | 72.16350893 | male |
| 4330 | 3.23 | 73.87471252 | female |
| 4332 | 1.7 | 70.54813273 | male |
| 4333 | N/A | 67.58295915 | male |
| 4334 | N/A | 68.65896397 | female |
| 4338 | 1.94 | 72.15803307 | female |
| 4347 | 1.97 | 78.46895192 | male |
| 4355 | 4.79 | 67.42689738 | female |
| 4356 | 3.8 | 79.5832877 | female |
| 4358 | 2.77 | 66.38922352 | male |
| 4367 | 2.23 | 68.47552294 | female |
| 4385 | 1.95 | 77.1903406 | female |
| 4386 | N/A | 76.78238966 | male |
| 4397 | 2.37 | 65.69105246 | male |
| 4398 | 2.34 | 79.9446939 | female |
| 4399 | 2.92 | 83.18639798 | female |
| 4402 | 3.53 | 67.63224181 | male |
| 4413 | 3.21 | 68.79586026 | female |
| 4414 | 1.73 | 71.60223415 | male |
| 4417 | 2.26 | 73.58996824 | male |
| 4419 | 1.45 | 74.69335232 | male |
| 4422 | 0.88 | 66.14828606 | male |
| 4426 | 2.67 | 82.22264812 | female |
| 4428 | 3.53 | 90.20370168 | male |
| 4439 | 3.29 | 65.85806593 | male |
| 4441 | 2.26 | 70.33731245 | female |
| 4444 | N/A | 70.14839558 | female |
| 4446 | N/A | 70.68502902 | male |
| 4448 | 2.85 | 84.9852152 | male |
| 4451 | 1.47 | 89.4507721 | female |
| 4454 | N/A | 81.0508159 | female |
| 4457 | 3.36 | 82.53750958 | female |
| 4471 | N/A | 77.46960902 | male |
| 4476 | 1.26 | 82.56488884 | male |
| 4481 | N/A | 85.02080824 | female |
| 4482 | N/A | 86.8251013 | male |
| 4483 | 2.66 | 88.88402147 | male |
| 4485 | 2.37 | 81.17949841 | male |
| 4486 | N/A | 87.74504435 | female |
| 4492 | 1.6 | 77.36282992 | female |
| 4502 | N/A | 70.38933304 | female |
| 4508 | N/A | 72.38528091 | male |
| 4509 | 2.43 | 80.41014128 | male |
| 4512 | N/A | 77.2012923 | female |
| 4516 | 2.76 | 75.97743949 | female |
| 4525 | 3.43 | 73.42295477 | male |
| 4534 | 3.67 | 76.11981163 | female |
| 4541 | 1.34 | 78.74000657 | female |
| 4543 | 2.82 | 80.88380243 | male |
| 4558 | 2.66 | 75.8186398 | female |
| 4560 | 2.82 | 94.73223086 | female |
| 4567 | 2.42 | 82.71273683 | female |
| 4570 | 3.09 | 66.09352754 | male |
| 4577 | 1.53 | 77.01511335 | female |
| 4582 | 3.63 | 68.79312233 | female |
| 4584 | 2.39 | 82.98379148 | male |
| 4588 | 2.91 | 87.26316942 | male |
| 4598 | 3.71 | 75.07118607 | male |
| 4600 | N/A | 70.47147081 | male |
| 4602 | N/A | 69.39546599 | female |
| 4606 | 2.88 | 69.36534881 | male |
| 4607 | 4.6 | 79.82148724 | male |
| 4610 | N/A | 73.18475523 | female |
| 4626 | 2 | 69.39272807 | male |
| 4627 | 2.61 | 82.5183441 | male |
| 4630 | N/A | 83.44102508 | female |
| 4638 | 4.57 | 65.95936918 | male |
| 4640 | 3.39 | 77.29438178 | female |
| 4650 | 2.37 | 70.8958493 | female |
| 4652 | 1.48 | 77.79542219 | male |
| 4655 | 3.21 | 68.83966707 | female |
| 4669 | 3.43 | 72.31957069 | female |
| 4670 | 2.38 | 72.10601248 | male |
| 4671 | 4.27 | 68.17708904 | male |
| 4674 | N/A | 83.63267988 | female |
| 4676 | 2.86 | 66.81086409 | female |
| 4680 | 2.15 | 92.45975249 | female |
| 4682 | 2.32 | 75.1067791 | male |
| 4684 | 2.56 | 74.0964845 | female |
| 4685 | 2.9 | 76.49490746 | female |
| 4688 | 1.51 | 90.23655678 | male |
| 4697 | N/A | 67.1421531 | female |
| 4700 | 1.67 | 69.26952141 | male |
| 4702 | 2.83 | 74.98357245 | male |
| 4706 | 1.21 | 78.44978644 | male |
| 4710 | 1.65 | 88.30084328 | female |
| 4712 | 4.28 | 82.73464024 | male |
| 4718 | 1.59 | 77.29985763 | female |
| 4720 | N/A | 76.76322418 | female |
| 4721 | 2.46 | 66.69039536 | female |
| 4731 | N/A | 71.08750411 | male |
| 4733 | 4.25 | 72.90274888 | male |
| 4735 | 3.43 | 69.80615486 | female |
| 4744 | 1.2 | 79.52852919 | female |
| 4746 | 4.51 | 65.81973497 | male |
| 4749 | 2.72 | 75.78030884 | female |
| 4752 | N/A | 94.50772095 | female |
| 4757 | N/A | 80.09801774 | female |
| 4762 | 2.24 | 72.09506078 | male |
| 4766 | 1.85 | 69.83627204 | male |
| 4776 | 2.4 | 66.33720294 | male |
| 4777 | 1.38 | 82.64155076 | female |
| 4783 | 1.48 | 67.28726317 | female |
| 4784 | 1.05 | 70.55087066 | male |
| 4785 | 2.95 | 74.86036579 | male |
| 4788 | N/A | 86.4527434 | male |
| 4792 | 1.5 | 67.5035593 | male |
| 4805 | 2.63 | 86.97842515 | female |
| 4808 | 2.73 | 77.78994634 | female |
| 4809 | N/A | 70.61658088 | female |
| 4815 | 2.87 | 79.42722593 | male |
| 4816 | 2.82 | 70.58920162 | female |
| 4817 | 2.86 | 71.38320009 | female |
| 4819 | 2.32 | 77.16296134 | female |
| 4822 | 3.92 | 75.38604753 | male |
| 4826 | 1.71 | 76.37717665 | male |
| 4839 | 2.33 | 67.91698609 | male |
| 4845 | 2.53 | 69.11345964 | male |
| 4847 | N/A | 82.05837258 | female |
| 4849 | 2.16 | 76.79060344 | male |
| 4862 | 3.32 | 76.27861132 | female |
| 4869 | 7.01 | 84.89760158 | female |
| 4873 | N/A | 86.57047421 | male |
| 4874 | 1.51 | 82.22264812 | female |
| 4875 | 4.46 | 83.52590078 | female |
| 4878 | 2.75 | 79.12331618 | female |
| 4879 | 4.13 | 90.42273574 | male |
| 4881 | 4.32 | 68.17161319 | male |
| 4883 | 3.32 | 76.36348702 | female |
| 4884 | N/A | 86.40619866 | female |
| 4896 | 4.02 | 66.80538824 | male |
| 4898 | N/A | 71.50093089 | male |
| 4900 | 3.76 | 84.6019056 | female |
| 4901 | 2.66 | 85.23710437 | female |
| 4906 | 2.97 | 70.29350564 | male |
| 4909 | 3.72 | 68.82323951 | female |
| 4911 | 1.78 | 70.38385719 | male |
| 4913 | 2.56 | 82.4964407 | male |
| 4914 | 3.1 | 79.62161866 | female |
| 4921 | 1.93 | 77.10820283 | female |
| 4934 | 3.26 | 72.99857628 | female |
| 4939 | 2.75 | 76.80703099 | female |
| 4940 | 3.7 | 75.20260651 | male |
| 4947 | 2.35 | 77.63114664 | female |
| 4962 | 2.28 | 76.25944584 | male |
| 4963 | 4.46 | 78.44157266 | female |
| 4965 | N/A | 89.31935166 | female |
| 4971 | 2.12 | 76.52228672 | male |
| 4975 | 2.75 | 81.34103603 | male |
| 4984 | 2.41 | 66.38374767 | male |
| 4988 | 6 | 69.44474866 | female |
| 4989 | 2.51 | 76.46479027 | male |
| 4990 | 3.54 | 71.5228343 | male |
| 4995 | 2.02 | 86.95652174 | male |
| 4996 | N/A | 93.69181908 | female |
| 4997 | 1.84 | 67.04906363 | male |
| 4998 | 3.52 | 87.47398971 | female |
| 5000 | 1.95 | 79.67363925 | male |
| 5002 | 3.51 | 89.69992334 | female |
| 5005 | 4.49 | 68.06209616 | female |
| 5007 | 2.47 | 69.16274231 | female |
| 5009 | N/A | 68.58503997 | male |
| 5011 | 4.15 | 74.12933961 | male |
| 5013 | 3.2 | 71.10119374 | female |
| 5020 | 2.77 | 78.91249589 | male |
| 5021 | N/A | 82.81404008 | male |
| 5025 | 2.78 | 70.92596649 | female |
| 5027 | 2.59 | 71.54473771 | male |
| 5028 | N/A | 68.10042712 | female |
| 5031 | N/A | 68.10864089 | male |
| 5032 | 2.02 | 79.498412 | female |
| 5043 | 1.07 | 66.82729164 | male |
| 5052 | 2.11 | 67.51177308 | female |
| 5053 | 2.79 | 70.57824992 | male |
| 5055 | 2 | 66.41386486 | female |
| 5057 | 2.62 | 66.88752601 | male |
| 5063 | 3.22 | 72.713832 | female |
| 5069 | 2.3 | 73.37914796 | female |
| 5070 | N/A | 73.82816778 | female |
| 5080 | 3.96 | 74.72073157 | male |
| 5082 | 1.58 | 80.01040412 | male |
| 5085 | 2.11 | 71.91435768 | female |
| 5088 | 4.31 | 88.69510459 | male |
| 5091 | 1.66 | 73.19570693 | female |
| 5106 | 1.67 | 73.63377505 | female |
| 5119 | 2.74 | 82.60869565 | female |
| 5123 | 1.03 | 71.12583507 | female |
| 5125 | 4.18 | 80.91118169 | female |
| 5126 | 1.18 | 72.45646698 | male |
| 5128 | 2.26 | 80.04873508 | female |
| 5132 | 2.26 | 77.32449896 | female |
| 5134 | 4.33 | 78.51823459 | female |
| 5142 | 2.47 | 67.13393933 | male |
| 5143 | N/A | 71.24356587 | female |
| 5159 | 3.63 | 67.1312014 | male |
| 5163 | 3.1 | 78.23622823 | female |
| 5170 | 2.9 | 66.53433359 | female |
| 5171 | N/A | 67.18595992 | female |
| 5173 | 2.55 | 86.85521849 | female |
| 5181 | 2.11 | 89.05651079 | female |
| 5186 | N/A | 69.15726645 | female |
| 5200 | 2.3 | 85.86682729 | male |
| 5207 | 1.61 | 76.46479027 | female |
| 5208 | N/A | 83.02759829 | male |
| 5213 | 4.63 | 94.97864418 | female |
| 5214 | 2.12 | 81.24520863 | male |
| 5216 | 2.83 | 70.37564341 | male |
| 5217 | 3.55 | 84.59642974 | female |
| 5222 | 2.02 | 75.94184646 | male |
| 5225 | 3.3 | 92.4926076 | male |
| 5228 | N/A | 73.95411236 | female |
| 5229 | 1.2 | 79.04665425 | female |
| 5232 | 1.86 | 88.61022889 | male |
| 5240 | 4.67 | 66.79443653 | female |
| 5253 | 4.02 | 74.04993977 | female |
| 5255 | 3.92 | 75.10130325 | male |
| 5262 | 2.36 | 84.09812726 | female |
| 5268 | N/A | 67.44880079 | female |
| 5269 | N/A | 72.47563246 | male |
| 5274 | 3 | 74.84941408 | female |
| 5279 | 1.82 | 67.43784909 | male |
| 5289 | 1.67 | 84.27335451 | female |
| 5291 | 2.52 | 67.73354507 | male |
| 5295 | 2.81 | 67.29821487 | male |
| 5302 | 2.21 | 73.2449896 | female |
| 5308 | 3.88 | 72.31683277 | male |
| 5309 | 1.76 | 70.93418027 | male |
| 5310 | 2.09 | 69.11345964 | male |
| 5311 | 1.53 | 67.9388895 | female |
| 5316 | 2.78 | 67.34749754 | female |
| 5318 | 1.26 | 71.80757858 | male |
| 5324 | 3.53 | 65.84711423 | male |
| 5338 | 2.66 | 76.29230095 | male |
| 5347 | 5.33 | 88.8347388 | female |
| 5357 | 2.19 | 69.67473442 | male |
| 5367 | 2.66 | 68.18530281 | male |
| 5369 | 2.65 | 66.16745154 | female |
| 5372 | N/A | 87.64921695 | female |
| 5381 | 1.49 | 93.82871537 | female |
| 5384 | 2.8 | 74.72620743 | male |
| 5389 | 2.84 | 71.92530939 | male |
| 5391 | 2.93 | 71.57485489 | female |
| 5392 | 2.42 | 66.43303034 | male |
| 5394 | N/A | 70.501588 | male |
| 5399 | 1.94 | 67.91151024 | female |
| 5403 | 2.6 | 66.31529953 | male |
| 5405 | 3.5 | 73.31891359 | female |
| 5422 | 1.57 | 80.11718322 | female |
| 5423 | 1.74 | 74.23338079 | female |
| 5427 | N/A | 81.48067024 | female |
| 5432 | 3.11 | 84.35001643 | female |
| 5439 | 1.98 | 70.0443544 | male |
| 5441 | 1.43 | 68.11411675 | female |
| 5442 | 2.82 | 74.41955974 | female |
| 5443 | 2.39 | 73.77888512 | female |
| 5454 | 1.81 | 82.65524039 | female |
| 5458 | 2.8 | 77.0096375 | male |
| 5462 | 2.61 | 82.62238528 | female |
| 5466 | 0.88 | 67.85948965 | male |
| 5469 | 3.94 | 70.65764976 | male |
| 5472 | N/A | 70.05804403 | male |
| 5476 | 2.91 | 81.06998138 | female |
| 5481 | 3.65 | 88.59106341 | female |
| 5493 | 2.32 | 66.84919505 | female |
| 5496 | N/A | 68.70550871 | female |
| 5504 | 2.18 | 89.85050925 | male |
| 5510 | 2.26 | 68.6836053 | female |
| 5513 | 2.46 | 78.15682839 | male |
| 5519 | 3.19 | 69.19559742 | male |
| 5529 | 3.74 | 80.28693462 | female |
| 5538 | 2.8 | 66.48778885 | male |
| 5542 | 2.5 | 86.93188041 | female |
| 5544 | 5.66 | 77.0945132 | female |
| 5546 | 1.45 | 83.77505202 | female |
| 5551 | 3.2 | 74.76727631 | male |
| 5553 | 2.39 | 76.6482313 | female |
| 5556 | 1.92 | 68.15792356 | male |
| 5558 | N/A | 69.5131968 | female |
| 5560 | N/A | 72.2675501 | male |
| 5561 | 2.74 | 69.60354835 | female |
| 5563 | N/A | 72.96298324 | female |
| 5572 | N/A | 78.12944913 | female |
| 5578 | 3.9 | 75.97743949 | female |
| 5579 | 3.21 | 75.18344103 | female |
| 5585 | N/A | 74.61395247 | female |
| 5588 | 1.89 | 73.70769905 | female |
| 5591 | 2.48 | 75.553061 | male |
| 5592 | N/A | 80.14730041 | female |
| 5593 | N/A | 75.99660497 | male |
| 5614 | 1.94 | 69.90198226 | female |
| 5615 | 3.32 | 68.45361954 | male |
| 5616 | 2.3 | 75.50104041 | male |
| 5620 | 3.69 | 79.01379915 | female |
| 5626 | 3.21 | 76.47300405 | female |
| 5637 | 1.45 | 87.20841091 | female |
| 5640 | 2.7 | 71.36677253 | male |
| 5642 | N/A | 93.96287373 | female |
| 5643 | 2.91 | 79.44091556 | male |
| 5644 | 4.08 | 77.09177527 | female |
| 5646 | N/A | 83.45197678 | male |
| 5652 | N/A | 83.98313438 | male |
| 5655 | N/A | 72.76037674 | male |
| 5693 | 2.35 | 69.41189355 | male |
| 5694 | N/A | 83.12068777 | female |
| 5698 | N/A | 71.39962764 | female |
| 5700 | 3.1 | 71.91435768 | male |
| 5704 | 3.49 | 88.73343555 | female |
| 5730 | 1.92 | 68.03745482 | male |
| 5755 | 4.3 | 103.0911182 | female |
| 5789 | N/A | 70.53991896 | male |
| 5792 | 1.63 | 67.66783485 | female |
| 5793 | 3.05 | 69.77603767 | female |
| 5846 | N/A | 90.74307305 | female |
| 5849 | 3.38 | 67.59117293 | male |
| 5850 | 1.58 | 80.33074143 | male |
| 5858 | 2.77 | 105.6729821 | female |
| 5876 | 3.83 | 90.26941189 | female |
| 5888 | 2.75 | 84.43489213 | male |
| 5900 | 3.08 | 67.00799474 | male |
| 5910 | 1.11 | 65.4939218 | female |
| 5912 | N/A | 66.24137553 | male |
| 5921 | 3.1 | 66.77800898 | female |
| 5935 | 2.51 | 69.11072172 | female |
| 5936 | 4.01 | 72.68645274 | male |
| 5937 | N/A | 65.46928047 | female |
| 5944 | 2.82 | 65.86080385 | male |
| 5968 | 2.97 | 69.03953565 | female |
| 5980 | 2.77 | 72.23743292 | male |
| 5992 | N/A | 79.91457672 | male |
| 5993 | 2.76 | 77.24236119 | female |
| 6004 | N/A | 66.86014675 | female |
| 6010 | 2.09 | 70.60015332 | female |
| 6011 | 2.18 | 74.34015989 | female |
| 6014 | N/A | 71.25999343 | female |
| 6029 | 3.3 | 72.47289454 | female |
| 6031 | 1.74 | 71.16964188 | male |
| 6037 | 2.21 | 81.00974702 | male |
| 6039 | 3.06 | 72.46468076 | female |
| 6046 | N/A | 72.78228014 | male |
| 6063 | 2.48 | 68.12506845 | male |
| 6064 | 3.09 | 71.48450334 | female |
| 6081 | 2.8 | 68.61789508 | male |
| 6083 | 3.05 | 76.60990034 | male |
| 6084 | N/A | 69.83900997 | female |
| 6085 | 2.1 | 66.51516811 | female |
| 6094 | N/A | 66.43576826 | female |
| 6096 | 1.51 | 73.30796189 | female |
| 6189 | 2.24 | 66.72051254 | female |
| 6197 | 2.62 | 76.57156938 | male |
| 6203 | 3.16 | 68.89168766 | male |
| 6224 | N/A | 84.10634104 | female |
| 6226 | 3.14 | 69.4064177 | female |
| 6232 | 4.29 | 86.78403242 | male |
| 6238 | 1.09 | 71.54473771 | female |
| 6253 | N/A | 67.65140729 | female |
| 6264 | N/A | 66.82181579 | female |
