# Supplementary figures and images for: Mitochondrial genomic variation associated with higher mitochondrial copy number: the Cache County Study on Memory Health and Aging
Source: BMC Bioinformatics. 2014 May 28;15(Suppl 7):S6. doi: 10.1186/1471-2105-15-S7-S6 (PMC4110732; doi:10.1186/1471-2105-15-S7-S6)

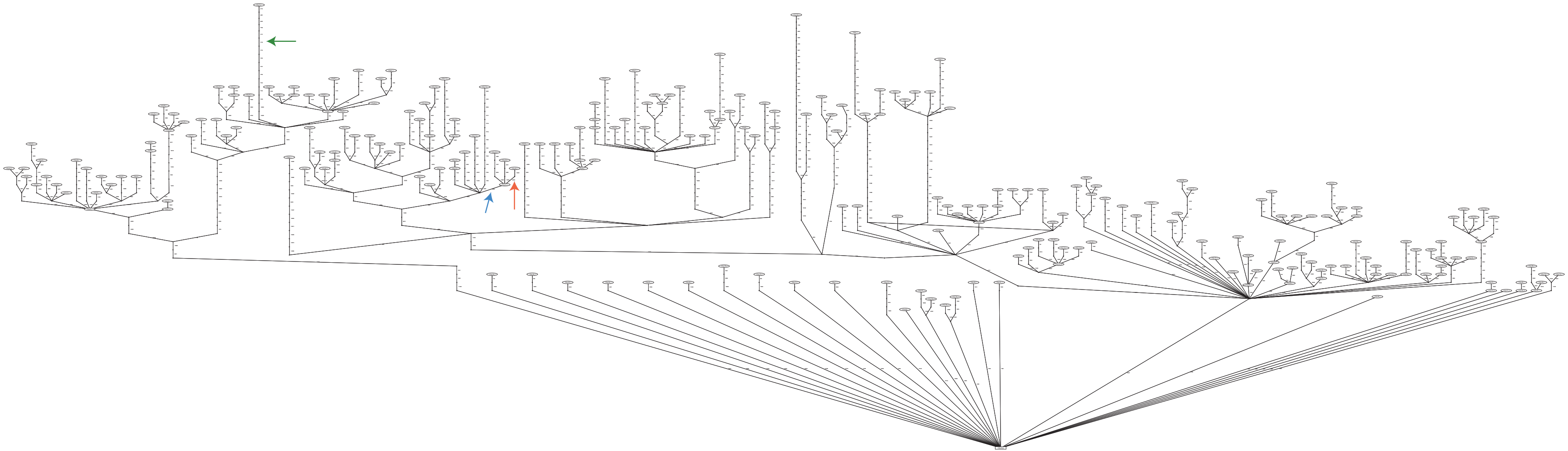

Supplement: Additional file 2 — (pdf) Haplotype network. Our haplotype network was constructed using TCS and 285 full mitochondrial genomes. The arrows point to each of the three branches representing the significant contrasts. The blue arrow points to branch 124, the red to branch 121, and the green to branch 50. [file 1471-2105-15-S7-S6-S2.pdf]

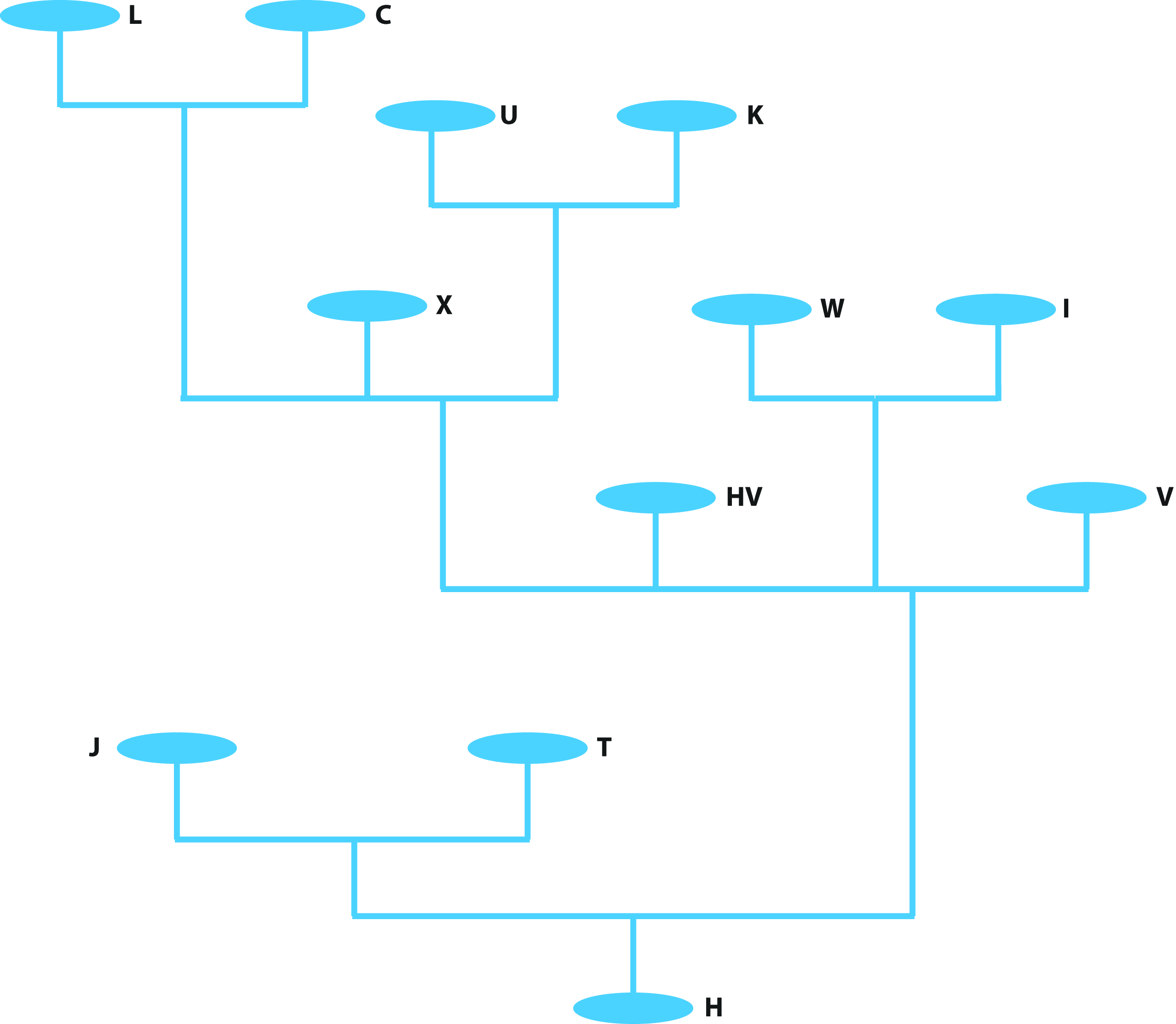

Supplement: Additional file 3 — (tiff) Haplotype network. We collapsed our haplotype network (Additional File 2) into nodes corresponding to the major mitochondrial haplogroups present in our network. [file 1471-2105-15-S7-S6-S3.tif]
