# Supplementary material for: Mitochondrial genomic variation associated with higher mitochondrial copy number: the Cache County Study on Memory Health and Aging
Source: BMC Bioinformatics. 2014 May 28;15(Suppl 7):S6. doi: 10.1186/1471-2105-15-S7-S6 (PMC4110732; doi:10.1186/1471-2105-15-S7-S6)
Supplement: Additional file 5 — (docx) Kinship coefficients for the clade defined by branch 124. We have listed the pairwise kinship coefficients for all the individuals in this clade. The IDs (row and column titles) correspond to the same IDs used in Additional File 1. [file 1471-2105-15-S7-S6-S5.docx]

|  | **3644** | **5310** | **4137** | **5308** | **3006** | **26** | **3903** | **5900** | **1322** | **3393** | **4161** | **59** | **4875** | **4869** |
| --- | --- | --- | --- | --- | --- | --- | --- | --- | --- | --- | --- | --- | --- | --- |
| **3644** | N/A | 0 | 0 | 0 | 0 | 0 | 0 | 0 | 0 | 0 | 0 | 0 | 0 | 0 |
| **5310** |  | N/A | 0 | 0.25 | 0 | 0 | 0 | 0 | 0 | 0 | 0 | 0 | 0 | 0 |
| **4137** |  |  | N/A | 0 | 0 | 0 | 0 | 0 | 0 | 0 | 0 | 0 | 0 | 0 |
| **5308** |  |  |  | N/A | 0 | 0 | 0 | 0 | 0 | 0 | 0 | 0 | 0 | 0 |
| **3006** |  |  |  |  | N/A | 0 | 0 | 0 | 0 | 0 | 0 | 0 | 0 | 0 |
| **26** |  |  |  |  |  | N/A | 0 | 0 | 0 | 0 | 0 | 0.25 | 0 | 0 |
| **3903** |  |  |  |  |  |  | N/A | 0.25 | 0.25 | 0 | 0.25 | 0 | 0.25 | 0.25 |
| **5900** |  |  |  |  |  |  |  | N/A | 0.25 | 0 | 0.25 | 0 | 0.25 | 0.25 |
| **1322** |  |  |  |  |  |  |  |  | N/A | 0 | 0.25 | 0 | 0.25 | 0.25 |
| **3393** |  |  |  |  |  |  |  |  |  | N/A | 0 | 0 | 0 | 0 |
| **4161** |  |  |  |  |  |  |  |  |  |  | N/A | 0 | 0.25 | 0.25 |
| **59** |  |  |  |  |  |  |  |  |  |  |  | N/A | 0 | 0 |
| **4875** |  |  |  |  |  |  |  |  |  |  |  |  | N/A | 0.25 |
| **4869** |  |  |  |  |  |  |  |  |  |  |  |  |  | N/A |
